# Defective Gpsm2/Gα_i3_ signalling disrupts stereocilia development and growth cone actin dynamics in Chudley-McCullough syndrome

**DOI:** 10.1038/ncomms14907

**Published:** 2017-04-07

**Authors:** Stephanie A. Mauriac, Yeri E. Hien, Jonathan E. Bird, Steve Dos-Santos Carvalho, Ronan Peyroutou, Sze Chim Lee, Maite M. Moreau, Jean-Michel Blanc, Aysegul Gezer, Chantal Medina, Olivier Thoumine, Sandra Beer-Hammer, Thomas B. Friedman, Lukas Rüttiger, Andrew Forge, Bernd Nürnberg, Nathalie Sans, Mireille Montcouquiol

**Affiliations:** 1 INSERM, Neurocentre Magendie, U1215, 146 rue Leo-Saignat, F-33077 Bordeaux, France; 2 Univ. Bordeaux, Neurocentre Magendie, U1215, F-33077 Bordeaux, France; 3 Section on Human Genetics, National Institute on Deafness and Other Communication Disorders, National Institutes of Health, Bethesda, Maryland 20892, USA; 4 Department of Otolaryngology, Hearing Research Centre Tübingen, Molecular Physiology of Hearing, University of Tübingen, D-72076 Tübingen, Germany; 5 Biochemistry and Biophysics Facility of the Bordeaux Neurocampus, F-33077 Bordeaux, France; 6 CNRS, Interdisciplinary Institute for Neuroscience, UMR 5297, 146 rue Leo-Saignat, F-33077 Bordeaux, France; 7 Univ. Bordeaux, Interdisciplinary Institute for Neuroscience, UMR 5297, F-33077 Bordeaux, France; 8 Department of Pharmacology and Experimental Therapy and Interfaculty Center of Pharmacogenomics and Drug Research, University of Tübingen, 72074 Tubingen, Germany; 9 UCL Ear Institute, London WC1X 8EE, UK

## Abstract

Mutations in *GPSM2* cause Chudley-McCullough syndrome (CMCS), an autosomal recessive neurological disorder characterized by early-onset sensorineural deafness and brain anomalies. Here, we show that mutation of the mouse orthologue of *GPSM2* affects actin-rich stereocilia elongation in auditory and vestibular hair cells, causing deafness and balance defects. The G-protein subunit Gα_i3_, a well-documented partner of Gpsm2, participates in the elongation process, and its absence also causes hearing deficits. We show that Gpsm2 defines an ∼200 nm nanodomain at the tips of stereocilia and this localization requires the presence of Gα_i3_, myosin 15 and whirlin. Using single-molecule tracking, we report that loss of *Gpsm2* leads to decreased outgrowth and a disruption of actin dynamics in neuronal growth cones. Our results elucidate the aetiology of CMCS and highlight a new molecular role for Gpsm2/Gα_i3_ in the regulation of actin dynamics in epithelial and neuronal tissues.

## Abstract (web summary)

Mutations in *GPSM2* cause a rare disease characterized by deafness and brain abnormalities. Here the authors show that Gpsm2 forms a molecular complex with a heterotrimeric G-protein subunit, whirlin and a myosin motor to regulate actin dynamics in neurons and auditory hair cell stereocilia.

Chudley-McCullough syndrome (CMCS, OMIM 604213) is a rare autosomal recessive neurological disorder in humans, characterized by early and severe onset of sensorineuronal deafness and hypoplasia of the corpus callosum (CC)^1^. CMCS syndrome patients often display frontal polymicrogyria (excessive small folds in the cortex of the brain) and heterotopia. These may be associated with cerebellar dysplasia, arachnoid cysts and ventriculomegaly. Some degree of delayed mental development has been reported for some patients, as well as occasional seizures, with overall psychomotor development generally normal^2^. The reason for this pleiotropy is not known and neither is the molecular basis of the pathology.

Recently, mutations in G-protein signalling modulator 2 (*GPSM2* in humans, also known as Leu-Gly-Asn repeat-enriched protein (*LGN*), mammalian Partner of inscuteable (*mPins*) *or Gpsm2* in mammals) were found to cause CMCS^2^,^3^,^4^. Because of the well-known role for Gpsm2 in the control of spindle position during orientated division^5^, it was suggested that CMCS might result from defects in asymmetric division of progenitors, both in the inner ear and the brain^2^. Recently, we demonstrated that Gpsm2 and one of its binding partner, the α-subunit of the heterotrimeric G-protein G_i3_ (Gα_i3_ encoded by *Gnai3*), control the asymmetric localization of the kinocilium in developing postmitotic hair cells (HCs) of the mammalian cochlea^6^,^7^. These results were confirmed by another group that also reported an accumulation of both proteins at the tip of the hair bundles of the HCs^8^,^9^.

A HC stereocilia bundle is an actin-rich organelle consisting of a specialized array of microvilli-derived structures that protrude from the apex of auditory and vestibular HCs. Mechanical deflection of the hair bundle gates mechano-sensitive ion channels in stereocilia that leads ultimately to afferent action potentials being conveyed to the central nervous system. Proper development and maintenance of stereocilia are vital for normal hearing^10^. Mechanisms that control HC bundle length are not fully understood. During early postnatal development, actin monomers are added at the barbed (plus) end of the stereocilium, resulting in elongation from the tip^11^,^12^,^13^. Several proteins are known to control this process^14^,^15^, with one common property being their localization at the tips of stereocilia during elongation^16^. The scaffold protein whirlin (encoded by *Whrn*) and myosin 15 (encoded by *Myo15*) are two of the best-characterized proteins among the HC bundle proteome participating in stereociliary growth. Myosin 15 interacts with whirlin and traffics it to the stereocilia tips where they colocalize with the actin-bundling and capping protein Eps8 (epidermal growth factor receptor pathway substrate 8)^17^,^18^,^19^, also dependant on myosin 15 for its localization at the stereociliary tip. Defects in all three genes individually cause abnormally short stereocilia^19^,^20^,^21^,^22^, deafness in mice^19^,^21^,^23^ and non-syndromic human deafness^24^,^25^,^26^.

In this study, we show that Gpsm2 and Gα_i3_ define an ∼200 nm domain at the tip of stereocilia, and that conditional deletion of either gene prevents stereocilia elongation, the likely cause of the early deafness and hearing deficits observed in mutant mice. Furthermore, we demonstrate that mutations identified in CMCS patients affect protein complexes, including a novel and functionally relevant interaction between Gpsm2 and whirlin. Using live super-resolution imaging, we show that actin dynamics are disrupted in growth cones of young hippocampal neurons from *Gpsm2* mutant mice, affecting neuronal outgrowth. These data support the idea of a global function for Gpsm2 in modulating actin dynamics. The versatility of Gpsm2/Gα_i3_ roles on actin and tubulin, in proliferative and postmitotic cells, is the probable cause of pleiotropy in CMCS brain anomalies.

## Results

### Gpsm2 and Gα_i3_ define a tip nanodomain within stereocilia

**Figure 1:**
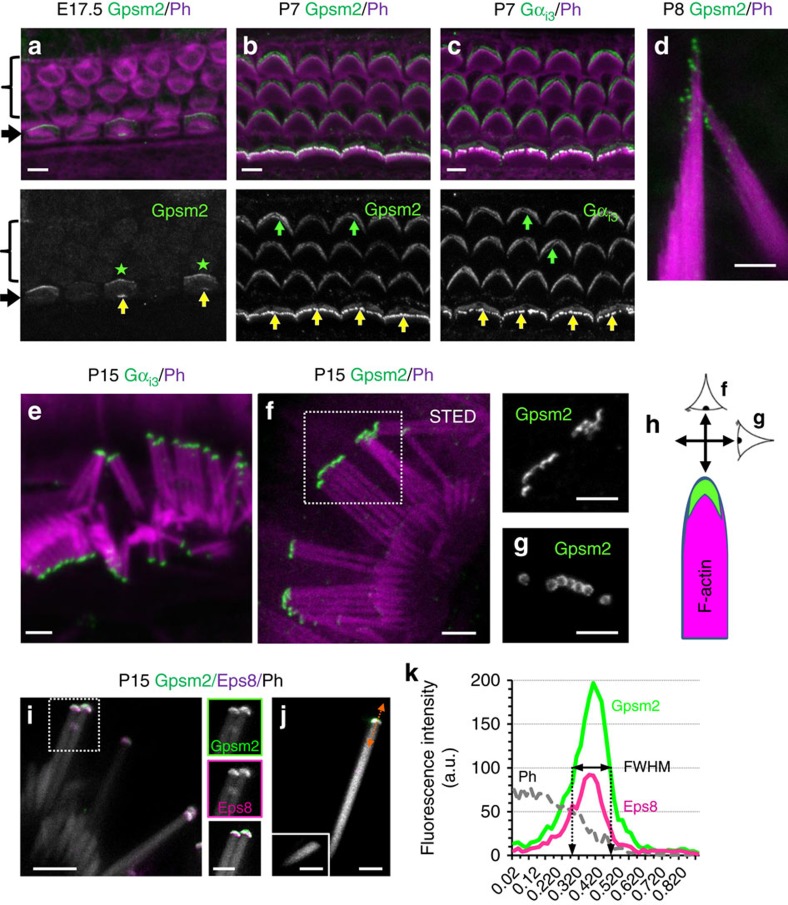
Gpsm2 and Gα_i3_ are dynamically expressed at the tips of stereocilia. (**a**–**c**) Surface view of whole mounts of rat cochlear sensory epithelium at E17.5 (**a**) and P7 (**b**,**c**) illustrating Gpsm2 (**a**,**b**, green) and Gα_i3_ (**c**, green) labelling in the actin-rich hair bundle labelled by phalloidin (Ph, magenta). (**a**) At E17.5, Gpsm2 localizes at the tips of stereocilia at the onset of hair bundle growth (yellow arrows), but also in an asymmetrical crescent in a distal region of the apical membrane of HCs (green asterisks). (**b**,**c**) By P7, both proteins accumulate at tips of stereocilia (green), strongly in IHC (yellow arrows) and more weakly in OHC (green arrows). Arrow: inner hair cell (IHC). Bracket: outer hair cell. Scale bars (**a**–**c**), 4 μm. (**d**) Gpsm2 (green) is localized at tips of P8 stereocilia of rat vestibular HC bundles. Ph: phalloidin. Scale bar, 2 μm. (**e**) At P15, confocal imaging reveals the accumulation of Gα_i3_ protein at the tip of individual stereocilium. Scale bar 2 μm. (**f**–**h**) At P15, STED super-resolution imaging of the Gpsm2-expression domain at an individual stereocilium tip (**f**). Gpsm2 accumulated at tips of IHC stereocilia (green), above the F-actin labelling (magenta). (**f**, right panel, **g**) Acquisition of single plane images in two perpendicular axis as illustrated on the schematic in **h** reveals the cap-like structure of the Gpsm2 nanodomain. Scale bars, 2 μm. (**i**) Triple STED labelling reveals two mostly overlapping nanodomains at stereocilia tips with Gpsm2 (green) and Eps8 (magenta) above the F-actin signal (Ph, white). Left images: individual channels for Gpsm2 (top) and Eps8 (middle). The bottom image illustrates the phalloidin channel (grey) with two-colour binary representation of Gpsm2 (green) and Eps8 (magenta), with the overlapping domain (plain white). Scale bar, 2 μm. (**j**) Isolated long (**j**) and short (**j**, inset) stereocilia illustrate the accumulation of the two proteins in the long stereocilium only. Scale bar, 1 μm. (**k**) Intensity profiles of phalloidin, Gpsm2 and Eps8 from (**j**) (orange line across tip domain). Immunostainings repeated more than six times.

We evaluated the localization of Gpsm2 and Gα_i3_ during the development of stereocilia hair bundles using previously characterized specific antibodies^6^. Gpsm2 was localized at the tip of the nascent hair bundle at embryonic day 17.5 (E17.5), the earliest phase of its formation (Fig. 1a, yellow arrows). Consistent with previous observations, the apical crescent-shaped accumulation of Gpsm2 was also present (Fig. 1a, stars; refs 6, 8). By postnatal day 7 (P7), when stereocilia are rapidly elongating, Gpsm2 and Gα_i3_ were enriched at the tips of the tallest row of inner hair cell (IHC) stereocilia, the actual sensory receptors receiving 95% of the fibres of the auditory nerve that project to the brain (Fig. 1b,c), but also in vestibular HCs of the ampulla (Fig. 1d). At P15, the enrichment is maintained in the tallest row, whereas we could not detect fluorescence in the middle and small rows (Fig. 1e,f). At this stage, the apical crescent-shaped staining became fragmented or absent, suggesting a gradual loss of these proteins from this zone. Multicolour STimulated Emission Depletion (STED) nanoscopy was used to probe the stereocilia tip compartment and revealed that Gpsm2 was concentrated into a circular cap-like structure (Fig. 1f–h), similar to what was described for myosin 15 (refs 12, 17, 27), above the actin core labelled with phalloidin. Multicolour STED revealed that both Gpsm2 and Eps8 domains mostly overlapped (Fig. 1i). To evaluate the size of the tip domain, we mechanically isolated stereocilia after immunocytochemistry, to obtain perfectly flat structures (Fig. 1j,k). Using fluorescence intensity line-scans along individual long stereocilia labelled with Gpsm2 and Eps8 antibodies and using the full-width at half-maximum (FWHM), we estimated that the tip domain extended ∼200 nm axially at the stereocilia tip (Gpsm2 FWHM=198±59 nm, *n*=10; Eps8 FWHM=200±63 nm, *n*=10). These results reveal a narrow stereocilia tip compartment of ∼200 nm where actin filament polymerization is regulated during hair bundle development.

### Loss of Gpsm2 or Gα_i3_ blocks stereocilia elongation

**Figure 2:**
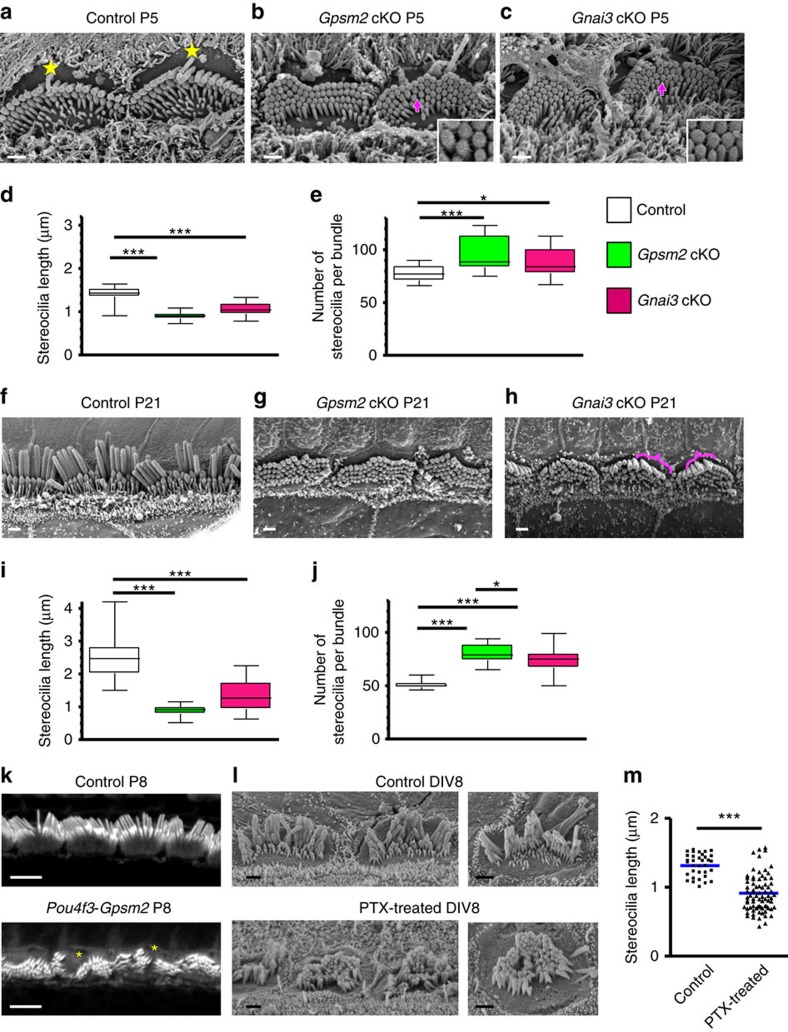
*Gpsm2* and *Gnai3* mutations inhibit stereocilia elongation. (**a**–**c**) SEM of cochlear inner hair cells (IHC) from controls (**a**), *Gpsm2* (**b**) or *Gnai3* cKOs (**c**) in P5 mouse. The kinocilium is indicated with yellow stars. In *Gpsm2* (**b**) and *Gnai3* (**c**) cKOs, the staircase pattern is almost absent with stereocilia of similar length and width. Lateral links between adjacent stereocilia are preserved (magenta arrows, insets). Scale bars, 1 μm. (**d**,**e**) Quantifications at P5 show reduced length of IHC tallest stereocilia and supernumerary stereocilia in both *Gpsm2* and *Gnai3* cKOs. Quantifications are presented as whisker box plots (min/max), *P<0.05; ***P<0.001 with one-way ANOVA (*post hoc* Bonferonni’s test). (**f**–**h**) SEM of basal cochlear IHC from controls (**f**), *Gpsm2* (**g**) or *Gnai3* cKOs (**h**) in P21 mouse. In *Gpsm2* cKOs, stereocilia are short, typically with more than four rows and severely reduced staircase pattern. A similar, but weaker phenotype was observed in *Gnai3* cKOs, with occasional longer stereocilia in a bundle with overall shorter stereocilia (**h**, magenta brackets). Scale bars, 1 μm. (**i**,**j**) Quantifications at P21 are consistent with the above illustrations with a severely reduced length of IHC stereocilia and supernumerary stereocilia in *Gpsm2* cKOs, and a similar but milder phenotype in *Gnai3* cKOs. Quantifications are presented as whisker box plots (min/max), *P<0.05; ***P<0.001 with one-way ANOVA (*post hoc* Bonferonni’s test). (**k**) 3D rendering of the surface of a control (top) and a *Pou4f3-Gpsm2* mutant (bottom) at P8. Mutants exhibit supernumerary rows of abnormally short stereocilia, sometimes split or fragmented (yellow asterisks). Scale bars, 4 μm. (**l**) SEM of postnatal cochlear explants treated or not for 8 days (DIV8) with 100 ng ml^−1^ PTX. In the PTX-treated samples (bottom panels), the stereocilia are shorter than in controls (upper pannels), with similar widths. Scale bars, 1 μm. (**m**) Quantification of the length of the tallest stereocilia cultures treated with PTX (blue line=mean). *n*=34 (control) and 76 stereocilia (PTX). Cultures repeated three times. ***P<0.0001 with unpaired Student’s *t*-test.

Owing to their localization at the site of actin polymerization, we hypothesized that Gpsm2 and Gα_i3_ might be molecular components required for the developmental elongation of stereocilia. To test this, we examined the cochlear sensory epithelium of conditional mutant mice generated with a Foxg1-Cre driver (hereafter, named *Gpsm2* cKO and *Gnai3* cKO), during critical stages of stereocilia elongation. At P5, in early phases of elongation, scanning electron microscopy (SEM) analyses revealed an ∼40% decrease in IHC tallest stereocilia length in *Gpsm2* cKOs (see Methods) and ∼25% decrease in *Gnai3* cKOs, compared with controls (Fig. 2a–d). There was also a statistically significant increase in the number of stereocilia per bundle in IHCs of *Gpsm2* cKOs (by ∼25%) and *Gnai3* cKOs (by ∼15%), with abnormal supernumerary (more than three) rows of short stereocilia compared with control (Fig. 2e). Omnidirectional lateral links could be seen in both *Gpsm2* and *Gnai3* cKOs (magenta arrows and insets in Fig. 2b,c), similar to the phenotype reported for the myosin 15-deficient mutant mouse *shaker 2* (ref. 28) (*sh2*, *Myo15*^*sh2*^). We conclude that Gpsm2 and Gα_i3_ are each required for the normal process of actin filament elongation that drives stereocilia development.

At P21, when elongation is completed, the average length of the tallest row of stereocilia was reduced by ∼70% and 50% compared with controls in *Gpsm2* and *Gnai3* cKOs, respectively (Fig. 2f–i, Supplementary Fig. 1a). The phenotype was more severe in *Gpsm2* cKOs that have short, thick stereocilia. In *Gnai3* cKO, we sometimes observed normal size stereocilia among an overall shortened hair bundle (Fig. 2h, magenta brackets), notably in the midbasal and more immature apical regions of the cochlea (Supplementary Fig. 1a, right panel, green arrows). In both cKOs, we observed supernumerary stereocilia per bundle, with ∼60% and 50% increase in *Gpsm2* and *Gnai3* cKOs, respectively, as compared with controls (Fig. 2j). These phenotypes are similar to those reported for *Myo15*, *Whrn* and *Eps8* mutants^20^,^21^,^22^,^29^. Our results show that IHC bundles in *Gpsm2* and *Gnai3* mutants share similar phenotypes, with stereocilia elongation significantly affected at the onset of hair bundle formation and an overall more severe phenotype in *Gpsm2* cKOs.

Owing to the well-documented role of Gpsm2 (Pins) on spindle orientation during asymmetric cell divisions, hearing loss in CMCS patients was proposed to result from a defect in planar cell polarity^2^,^3^,^4^. Once apical/basal polarity has been established in mice, formation of the stereocilia bundle initiates in postmitotic HCs in the basal part of the cochlea coil. The critical phase of stereocilia elongation required to form the mature hair bundle occurs mostly after birth (P0). To confirm a postmitotic role for Gpsm2 in hair bundle elongation, we generated *Gpsm2* conditional mutants using the *Pou4f3* promoter to drive Cre-mediated recombination in postmitotic HCs only^30^ (Supplementary Fig. 1b). Hair bundles in these mutants displayed supernumerary rows of shorter stereocilia at P8 (Fig. 2k), similar to the phenotype we observed with an early embryonic deletion of the gene. To confirm a postmitotic function of Gα_i_ in stereocilia elongation, we treated cochlear explants from newborn rats (P0/P1) for 8 days *in vitro* (DIV) with increasing concentrations of pertussis toxin (PTX), a pharmacological inhibitor of all three Gα_i_ protein isoforms (Supplementary Fig. 1c). After 8 DIV, we found that stereocilia elongate in control cultures, whereas in the presence of PTX, hair bundles of IHCs exhibited supernumerary rows of short stereocilia (Fig. 2l), similar to those observed for the *Gnai3* cKOs. In these PTX-treated samples, the average length of the tallest row of stereocilia was reduced by ∼30% when compared with controls (Fig. 2m).

### Loss of Gpsm2 or Gα_i3_ causes hearing and balance deficits

**Figure 3:**
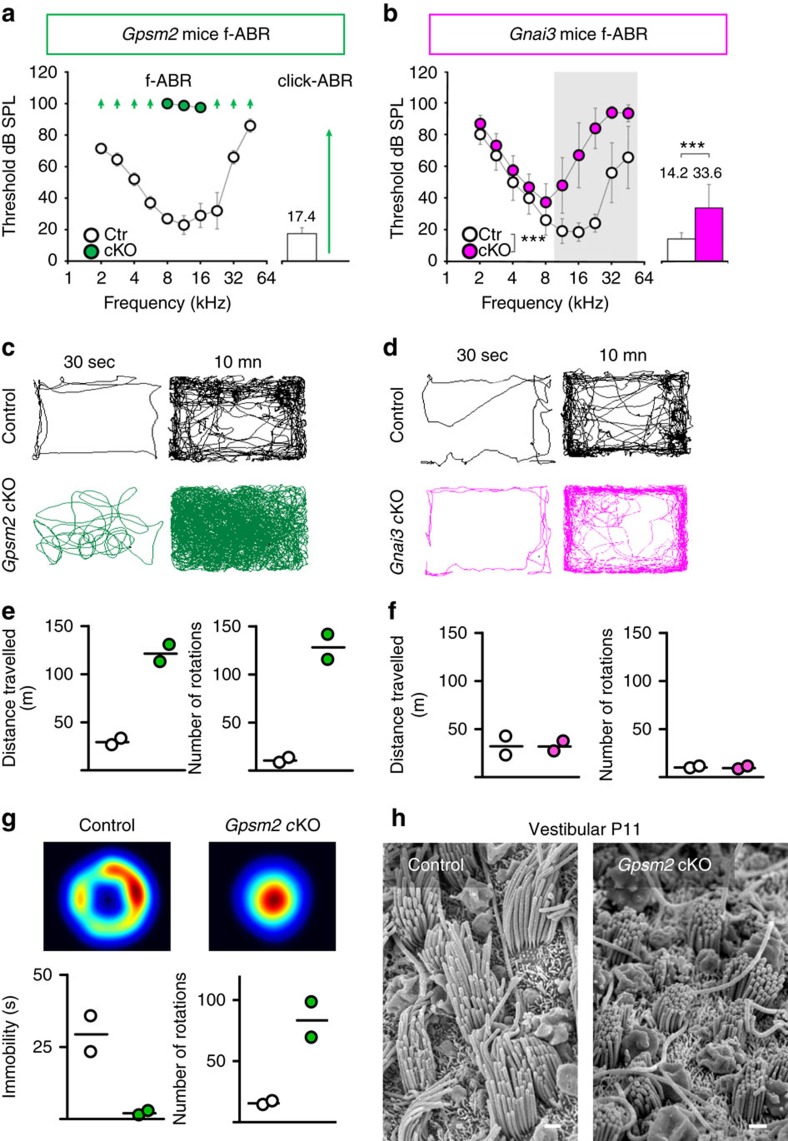
*Gpsm2* and *Gnai3* mutations affect cochlear and vestibular function. (**a**,**b**) Hearing tests on 4-week-old mice reveal severe threshold increases in *Gpsm2* cKO (**a**), compared with high frequency loss only in *Gnai3* cKOs (**b**). Arrow in **a** indicates ABR thresholds exceeding the maximum testable intensity. Mean±s.d. click-evoked ABR (click-ABR) and tone-burst-evoked ABR (f-ABR). Mean threshold values (in dB SPL) of click-ABR of control mice are shown above corresponding bars. ***P<0.001 (Grey shaded area: *P*<0.05) by two-way ANOVA (*post hoc* Bonferonni's multi comparisons test). f-ABR: Control (Ctr) and cKO, *n*=8 ears from eight mice click-ABR: Control and cKOs: *n*=16 ears from eight mice. (**c**,**d**) Left panel: *Gpsm2* cKOs (green traces) display increased circling activity in a representative open-field during the first 30 s and at the end of the track (10 min) compared with control littermates, whereas *Gnai3* cKOs (magenta traces) are unaffected (right panel). (**e**,**f**) *Gpsm2* cKOs mice cover more distance and rotate more than *Gnai3* cKOs mice (each circle is an individual mouse). Open white circles are controls. (**g**) Top: heat map of force swim test occupancy for control and *Gpsm2* cKOs. Bottom: during the 2 min test, *Gpsm2* cKO mice showed less immobility and more body axis rotation compared with controls. (**h**) SEM of the surface view of the macula of the utricle of P11 mice in control (left) and *Gpsm2* cKO (right). Stereocilia elongation in the mutant is dramatically reduced compared with control. Scale bars, 1 μm.

We assessed the auditory function of *Gpsm2* and *Gnai3* cKOs by measuring the threshold of auditory brainstem responses (ABR) using click-tones and pure tone of frequencies from 1 to 45 kHz. *Gpsm2* cKOs were profoundly deaf at 6 weeks of age (Fig. 3a), with ABR thresholds above 90 decibel sound pressure level at all frequencies, whereas *Gnai3* cKOs displayed specifically high frequency hearing loss starting at 11.3 kHz at the same age (Fig. 3b). These results are consistent with the differences in the severity of the phenotype we observed morphologically, and demonstrate that Gpsm2 and Gα_i3_ are essential for stereocilia maturation and hearing. Another *Gpsm2* cKO resulting in a truncated protein lacking the carboxy terminus was recently reported to be deaf^31^. Also, *Gpsm2,* but not *Gnai3* cKOs, exhibited increased hyperactive behaviour (413%) from controls and circling behaviour (>130 rotations in cKO) (Fig. 3c–f). *Gpsm2* cKOs swam in tight circles and are more mobile than their control littermates in a forced swim test (Fig. 3g). These results are indicative of a vestibular dysfunction and are consistent with the shortened stereocilia observed in the vestibular epithelium of the *Gpsm2* cKOs (Fig. 3h).

### Gpsm2/Gα_i3_ need myosin 15/whirlin for stereocilia elongation

**Figure 4:**
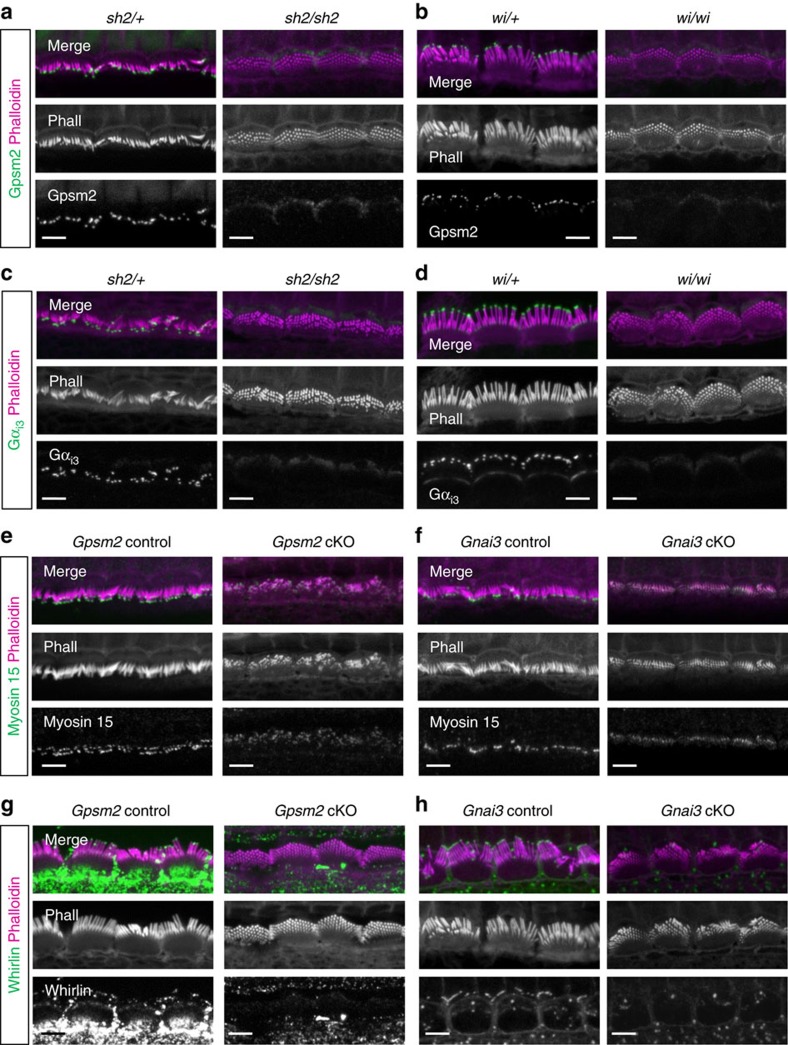
Gpsm2 and Gα_i3_ localization at stereocilia tips is dependent on myosin 15 and whirlin. (**a**–**d**) Immunocytochemistry for Gpsm2 (**a**,**b**) or Gα_i3_ (**c**,**d**) and staining with phalloidin (Ph, magenta) shows protein localization at the tips of stereocilia in IHCs from *shaker* 2 (*sh2/+*, **a**,**c**) and *whirler* (*wi/+*, **b**,**d**) control mice at P8. Homozygous mutations (*sh2/sh2* and wi*/wi*) lead to a loss of Gpsm2 and Gα_i3_ stereocilia tip staining. The immunostainings were repeated four times. (**e**,**f**) Immunocytochemistry for myosin 15 (green) and staining with phalloidin (Ph, magenta) shows protein localization at tips of stereocilia in IHCs from controls of *Gpsm2* (**e**) and *Gnai3* (**f**) cKO P8 mice. Myosin 15 protein is still present at the tip of shortened stereocilia of both cKOs, though in reduced amounts. (**g**,**h**) Immunocytochemistry for whirlin (green) and staining with phalloidin (Ph, magenta) shows protein localization at tips of stereocilia in IHCs from controls of *Gpsm2* (**g**) and *Gnai3* (**h**) P8 mutant mice*. Gpsm2* and *Gnai3* mutation leads to a strong reduction of whirlin stereocilia staining. Scale bars, 4 μm. The immunostainings (**e**–**h**) were repeated six times and imaged blindly.

**Figure 5:**
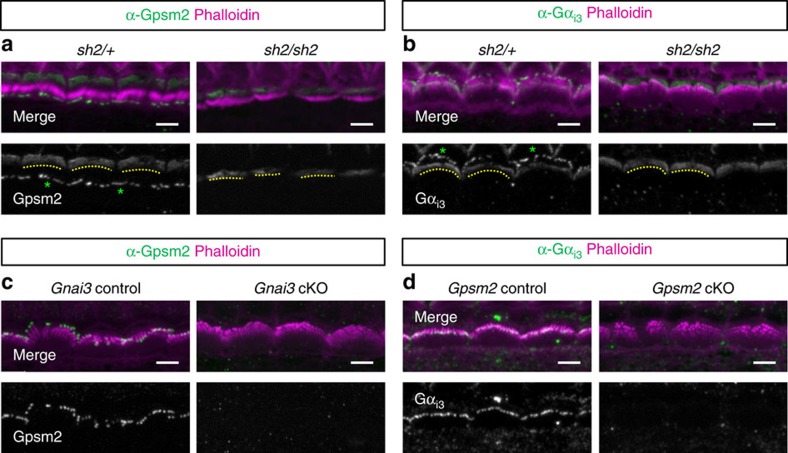
Gpsm2 and Gα_i3_ depend upon myosin 15 to reach stereocilia tips but not for their targeting at HC apical membrane. (**a**,**b**) Immunocytochemistry for Gpsm2 (**a**) or Gα_i3_ (**b**) shows both protein localization at the tips of stereocilia (green asterisks), and at the apical membrane of HC as a crescent-shape (yellow dashed-line) from controls of *shaker* 2 (*sh2/+*). Homozygous mutation (*sh2/sh2*) leads to a loss of Gpsm2 and Gα_i3_ stereocilia tip staining, whereas the apical crescent is maintained. (**c**) Immunocytochemistry for Gpsm2 (green) shows protein localization at the tips of stereocilia in IHCs from controls of *Gnai3*, but absent from *Gnai3* cKO. (**d**) Reciprocally, immunocytochemistry for Gα_i3_ (green) shows protein localization at the tips of stereocilia in IHCs from controls of *Gpsm2*, but absent from *Gpsm2* cKO. Scale bars, 4 μm. The immunostainings were repeated four times.

Since *Gpsm2* and *Gnai3* mutants have short stereocilia similar to *shaker 2* (*sh2*; myosin 15 functional null) and *whirler* (*wi*) mutants, we hypothesized that these proteins form a larger macromolecular complex. The delivery of whirlin to stereocilia tips requires functional myosin 15, whereas trafficking of myosin 15 can occur independently of whirlin^17^. We found that Gpsm2 (Fig. 4a,b) and Gα_i3_ (Fig. 4c,d) were both absent from the stereocilia tips of *sh2/sh2* (Fig. 4a,c) and *wi/wi* HCs at P8 (Fig. 4b,d). These data demonstrate that Gpsm2 and Gα_i3_ require myosin 15 to be trafficked to the tips of stereocilia. Myosin 15 was still present at the tip of short stereocilia of *Gpsm2* and *Gnai3* cKOs at P8 (Fig. 4e,f), whereas whirlin localization was absent in *Gpsm2* and *Gnai3* cKOs (Fig. 4g,h). Importantly, in earlier stages (P4) we observed that the apical crescent of Gpsm2 and Gα_i3_ was maintained in s*h2/sh2* mice (Fig. 5a,b). This result demonstrates that both proteins depend upon different interactions and protein complexes for apical membrane or stereocilia tip traffic within the HC. We also confirmed that the localization of both proteins at the tips of stereocilia was interdependent (Fig. 5c,d) as is the case in many other systems^32^,^33^. Altogether, our data demonstrate that Gpsm2 and Gα_i3_ are sorted to the apical membrane and to the stereocilia tip via different protein interactions.

Our data suggest that whirlin is necessary for the trafficking and/or the maintenance of Gpsm2 and Gα_i3_ at the tip of the stereocilia, and absence of whirlin in *Gpsm2/Gnai3* cKOs also suggest that the protein module participates in whirlin maintenance at the stereocilia tips. Altogether, our data show that the similar phenotypes of *sh2, wi, Gpsm2, Gnai3* mutant mice arise from a common molecular function in driving stereocilia elongation.

### Gpsm2 and whirlin interact

**Figure 6:**
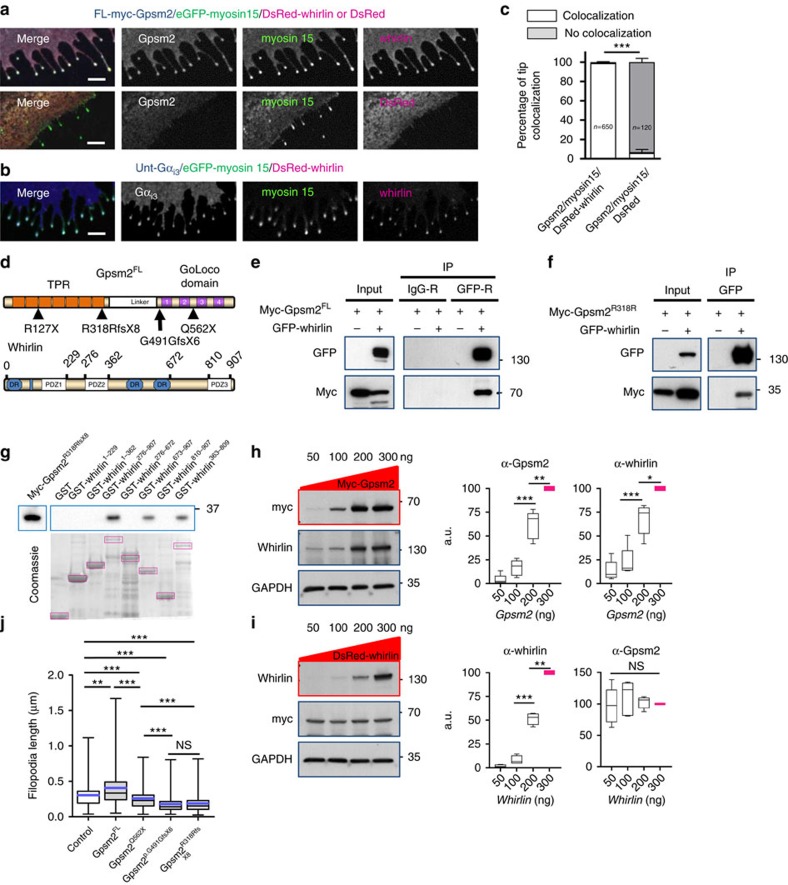
New protein complex between Gpsm2 and whirlin. (**a**–**c**) COS-7 cells transiently transfected with GFP–Myo15, DsRed-whirlin, myc-Gpsm2^FL^, untagged Gα_i3_ or DsRed as indicated. (**a**) Co-expression of myosin 15 and whirlin leads to the formation of actin-rich filopodial structures with Gpsm2 accumulating at the tip with the two proteins. Absence of whirlin (DsRed only) markedly reduces the colocalization at the tips. (**b**) Gα_i3_ also accumulates at the tip of filopodia with myosin 15 and whirlin. Transfections were repeated more than three times. Scale bars, 4 μm. (**c**) Percentage of filopodia tips (±s.e.m.) displaying colocalization of Gpsm2, myosin 15 and whirlin in the two contexts. *n*=number of filopodia tips from three independent experiments. ***P<0.001 with Mann–Whitney test. (**d**) Schematic representation of Gpsm2^FL^ and whirlin proteins. Four variants identified in CMCS patients, including Gpsm2^R318RfsX8^ are indicated. (**e**) Whirlin co-immunoprecipitates with myc-Gpsm2^FL^ but not with non-immune IgG. (**f**) The interaction is maintained, though reduced, with the Gpsm2^R318RfsX8^ (Gpsm2^R318R^) variant. Representative images of *n*>3 independent experiments. (**g**) A GST-pull-down assay indicates that the unstructured region of whirlin between aa 672 and aa 810 binds to Gpsm2^R318RfsX8^. Representative image of *n*=3 independent experiments. (**h**,**i**) Increasing amounts of myc*-*Gpsm2^FL^*-*encoding cDNA over constant amounts of whirlin leads to a net increase in whirlin expression levels, whereas increasing doses of whirlin had no significant effect on Gpsm2 levels. Five independent experiments presented as whisker box plots (min/max) combined with dot plot (blue bars representing the mean values) ***P<0.001, **P<0.01, *P<0.05 (for **h**,**i**) with one simple *t*-test or one-way ANOVA (post-hoc Bonferonni’s test). NS, not significant. (**j**) Gpsm2^FL^ significantly increased the length of filopodia generated by eGFP–myosin 15 and DsRed-whirlin (control) in COS-7 cells. Constructs bearing CMCS mutations led to shorter filopodia, compared with control or to Gpsm2^FL^. Data are presented as whisker box plots (min/max) from three independent experiments (*n*=220, 207, 345, 228 and 188 filopodia, in the order indicated on the histogram’s *x* axis) (blue bars represent the mean values). ***P<0.001, **P<0.01 with Kruskal–Wallis test (post-hoc Dunn's Multiple Comparison Test). NS, not significant.

To further explore the functional interactions between myosin 15, whirlin, Gpsm2 and Gα_i3_ proteins, we used a heterologous system. COS-7 cells co-transfected with complementary DNA (cDNA) constructs encoding both myosin 15 and whirlin result in numerous actin-rich filopodia protrusions and the two proteins accumulate at the tips of those extensions^17^. Using this system, we show that Gpsm2 and Gα_i3_ were also transported to the tip of the filopodia in the presence of myosin 15 and whirlin (Fig. 6a,b). The majority of filopodia tips (99%) contained Gpsm2 in the presence of whirlin and myosin 15, and this number drops to 6% when an empty vector coding for DsRed replaces DsRed-whirlin (Fig. 6c). Co-immunoprecipitation experiments confirmed an interaction between full-length myc-tagged Gpsm2^FL^ and either green fluorescent protein (GFP)–whirlin or untagged whirlin, whereas non-immune IgG or pEGFP–C3 coding for GFP did not co-immunoprecipitate either myc-Gpsm2 nor whirlin demonstrating the specificity of the interaction (Fig. 6d,e, Supplementary Fig. 2a,b). To assess the interaction in the context of CMCS, we evaluated a human *GPSM2* variant reported in patients that is predicted to truncate the GoLoco and linker domains, and is associated with multiple and severe anatomical brain abnormalities^2^. The Gpsm2^R318RfsX8^ variant still bound to whirlin, indicating that the N-terminal domain of Gpsm2 was sufficient for this interaction (Fig. 6d,f). We used glutathione *S*-transferase (GST)-pull down assays to show that the unstructured C-terminal region of whirlin, between aa 672 and aa 810 was the minimal domain required to interact with Gpsm2^R318RfsX8^ (Fig. 6d,g). All of the whirlin GST-constructs lacking this region failed to interact with myc-Gpsm2^R318RfsX8^, whereas the three GST-constructs containing this region pulled down the variant (Fig. 6g, lanes 4, 6, 8). To test if Gpsm2^FL^ may act as an adapter stabilising whirlin at the tips of stereocilia, we transfected HEK293T cells with increasing amounts of myc-Gpsm2^FL^-encoding cDNA while maintaining the quantity of DsRed-whirlin cDNA constant. Under these conditions, we observed a net increase in DsRed-whirlin protein expression levels, whereas increasing doses of DsRed-whirlin had no significant reciprocal effect on myc-Gpsm2 levels (Fig. 6h,i). Controls with increasing amount of cDNA encoding myc-Gpsm2^FL^ did not affect DsRed levels (Supplementary Fig. 2c). One possible interpretation of these results is that when in a complex with Gpsm2, whirlin protein may be stabilized, possibly by being less susceptible to degradation.

As Gpsm2^R318RfsX8^ still bound whirlin, we evaluated if it could modulate the ability of myosin 15 and whirlin to generate filopodia. Figure 6d illustrates the position of GPSM2 truncating variants identified in CMCS patients and the resulting predicted truncated proteins. In the presence of myc-Gpsm2^FL^, 42% of COS-7 cells extended filopodia, whereas in the presence of Gpsm2^R318RfsX8^, this number was reduced by half to 21% (Supplementary Fig. 2d, see Methods). These results suggest that the truncation of the linker and GoLoco domains in Gpsm2^R318RfsX8^ impairs the filopodia-generating ability of the myosin 15/whirlin complex. To evaluate if some of these truncation mutations could affect the proteins levels, we quantified immunoblots of the different Gpsm2 variants. Results show that the myc-Gpsm2^Q562X^ variant (missing the last two GoLoco domains) had an ∼15±4% reduction in protein levels compared with the myc-Gpsm2^FL^, whereas myc-Gpsm2^p.G491GfsX6^ variant (missing all four GoLoco domains) led to an ∼70±4% decrease, and the Gpsm2^R318RfsX8^ variant (missing all four GoLoco domains and the linker domain) led to ∼60±4% decrease (Supplementary Fig. 2e). The shortest truncation (Gpsm2^R127X^), with only two tetratricopeptide repeats domains remaining, was the most severe (too weak for quantification). The impact of three of these four truncations was evaluated upon filopodia length in the presence of myosin 15 and whirlin. We first noted that co-expressing Gpsm2^FL^ with myosin 15 and whirlin statistically increased the average length of filopodia (Fig. 6j). This increase was lost with all of the Gpsm2 variants tested. The truncated proteins also affected the myosin 15/whirlin effect, suggesting a dominant-negative effect. Notably, the shortest truncations retaining a amino-terminus (Gpsm2^p.G491GfsX6^, Gpsm2^R318RfsX8^) had the most severe effect on myosin 15/whirlin elongation. Taken together these data show that truncation mutations lead to some reduction in Gpsm2 protein levels, but they also indicate that the shortest forms still harbouring a N-terminus are able to interact with whirlin, therefore affecting the ability of myosin 15 and whirlin to induce filopodia elongation. We conclude that Gpsm2 is a new binding partner of whirlin, and that CMCS mutations affect the ability of the myosin 15/whirlin complex to generate filopodia.

### *Gpsm2* mutation affects neuronal development and motility

**Figure 7:**
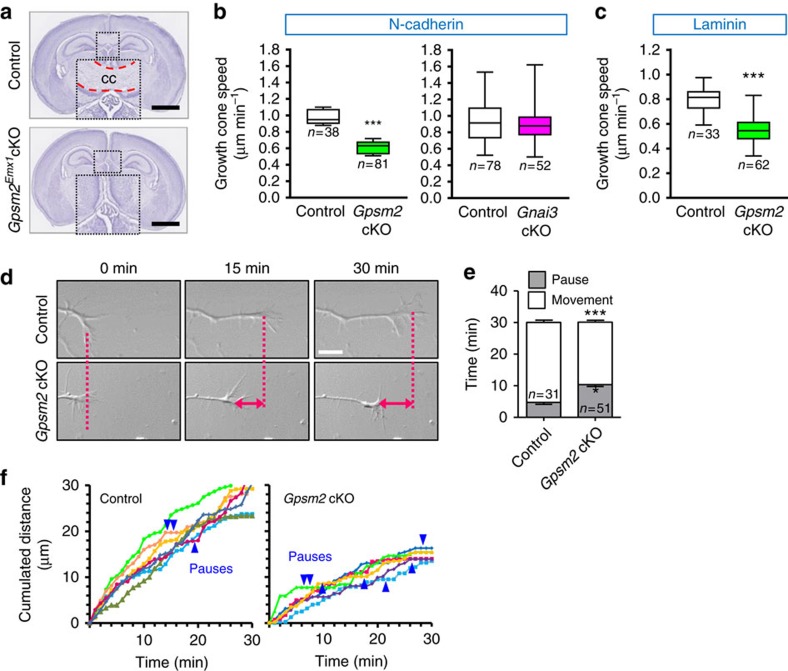
*Gpsm2* mutation leads to hypoplasia of the corpus callosum and affects growth cone outgrowth. (**a**) Hematoxylin staining of coronal sections from P6 *Gpsm2*^*Emx1*^ control (upper panel) and cKO brains (lower panel) at a caudal levels. Note the abscence of corpus callossum (CC) in the cKO mouse (inset). Scale bar, 1 mm, *n*=4 independent experiments. (**b**,**c**) In cultured hippocampal neurons at DIV2, N-cadherin-dependent outgrowth was reduced in *Gpsm2* cKOs, but not in *Gnai3* cKOs (**b**). The reduction in outgrowth was maintained on a laminin substrate in *Gpsm2* cKOs (**c**). Data from three to six independent experiments are presented as whisker box plots (min/max) (*n*=number of growth cones). ***P<0.001 with unpaired Student’s *t*-test. (**d**) Images from three time points (0, 15 and 30 min) of a time-lapse movie from 2 DIV control and *Gpsm2* cKO hippocampal neurons plated on N-cadherin-coated glass and showing the difference in distance covered (dotted lines and double-headed arrows). Scale bar=10 μm. (**e**) Quantification of the average number of pauses (±s.e.m.) during a 30 min period of growth cones from control (*n*=31 neurons) compared with *Gpsm2* cKO (*n*=51 neurons), from four independent experiments. The *Gpsm2* cKO growth cones pause more than controls. ***P<0.001, *P<0.05 with unpaired Student’s *t*-test. (**f**) Cumulated distance covered by control and *Gpsm2* cKO growth cones over a 30 min period. Some pauses are indicated with blue arrows.

In addition to early-onset sensorineural deafness, patients with CMCS also display specific brain malformations on magnetic resonance images; hypoplasia of the CC being a hallmark of this pathology^1^. To evaluate the impact of Gpsm2 on CC development, we generated *Emx1*-Cre*Gpsm2 cKOs (Gpsm2^Emx1^), deleting *Gpsm2* in the early and dorsal telencephalon. Analysis of brains from these cKOs confirmed the existence of a caudal CC agenesis (Fig. 7a), as reported in CMCS patients. CC defects can result from a disruption in neuronal progenitors or defects in axonal elongation and guidance, which are dependent upon the microtubule and actin cytoskeleton^34^. Neurite elongation is due to the motility of the growth cone driven in part by protrusive forces generated by actin polymerization at the leading edge^34^,^35^ and the existence of a retrograde flow of actin resulting from a balance of filament polymerization and depolymerization, among other factors^36^,^37^. We therefore hypothesized that actin dynamics could be impaired in mutant *Gpsm2* neurons and affect outgrowth. Measures of growth cone locomotion on N-cadherin-coated substrates showed that outgrowth of *Gpsm2* cKOs neurons was reduced by 37% compared with controls (control 0.97±0.04 μm min^−1^ versus cKO 0.61±0.02 μm min^−1^), whereas the speed of growth cones from *Gnai3* cKOs was indistinguishable from controls (control 0.92±0.02 μm min^−1^; cKO 0.89±0.02 μm min^−1^) (Fig. 7b). This *Gpsm2* cKO phenotype was also observed on a laminin-coated substrate, suggesting that the outgrowth reduction was not specific to an N-cadherin substrate (Fig. 7c). The number of pauses the growth cone made during the 30 min time-lapses on N-cadherin substrate was increased in *Gpsm2* cKO neurons (Fig. 7d–f, Supplementary Movie 1).

**Figure 8:**
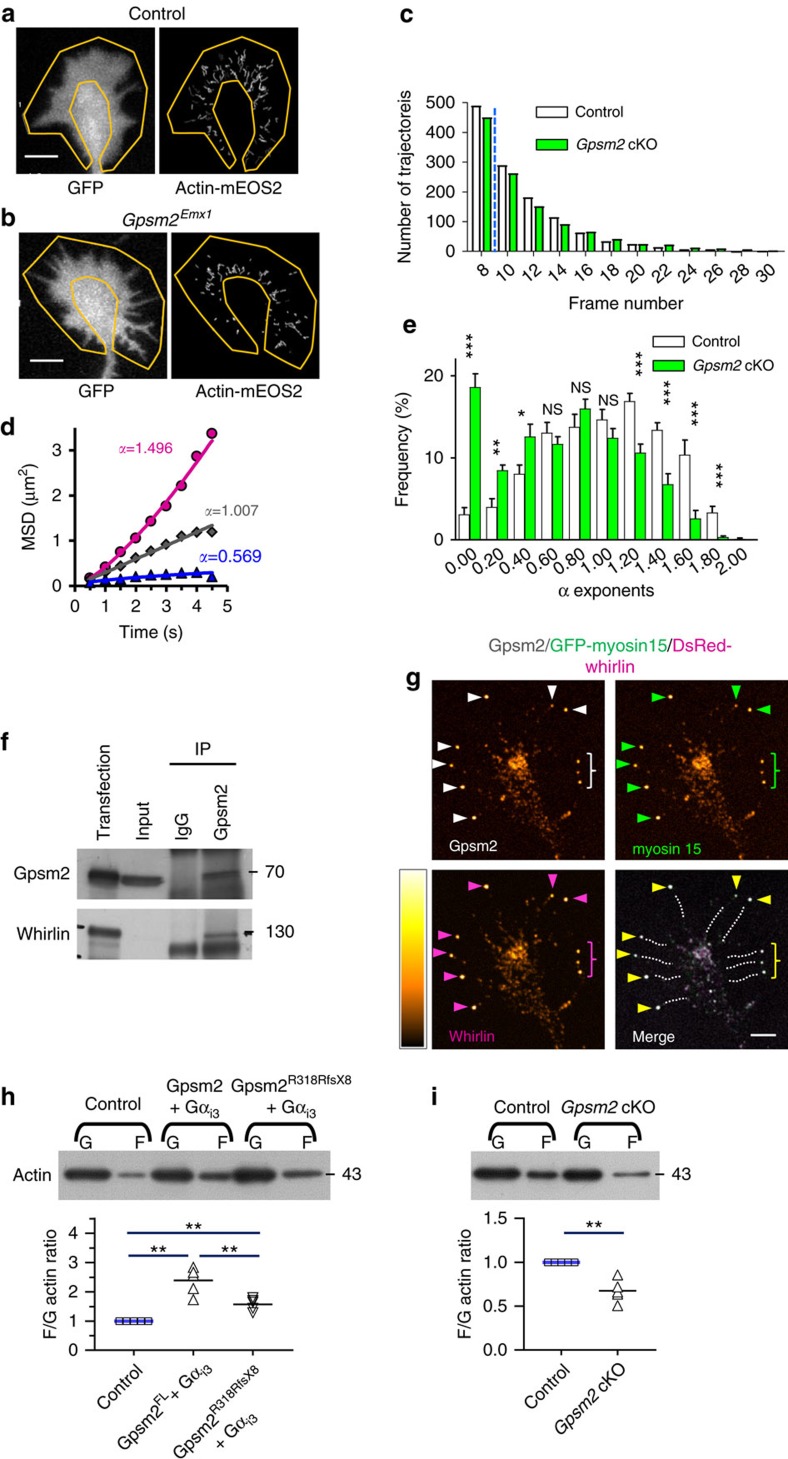
Gpsm2 co-immunoprecipitates whirlin in brain lysates and increases actin polymerization. (**a**,**b**) Representative DIV2 growth cones from a control (**a**) and a *Gpsm2* cKO (**b**) with the outlined peripheral region where sptPALM data were collected (yellow) and the corresponding individual actin-mEOS2 trajectories (3 min recording). Note the overall more-confined behaviour of the actin molecules in the cKO. Scale bars, 5 μm. (**c**) Distribution of the actin-mEOS2 trajectory length shows a median trajectory length of nine frames for both genotypes (blue line). (**d**) Representative mean squared displacement (MSD) over time for each of the three types of actin-mEOS2 behaviours with their corresponding α values. The plain curves represent fits to the function MSD=4Dt^α^, where D is a diffusion coefficient and *α* is a power law exponent. (**e**) Repartition of the α values of actin-mEOS2 molecules in control and *Gpsm2* cKO neurons on N-cadherin substrate. Values from 11 (control) and 12 (cKO) growth cones from three separate experiments (±s.e.m., *n*=1344 trajectories for control and 1121 for mutant). ***P<0.001, **P<0.01, *P<0.05 with an unpaired Student’s *t*-test or Mann Whitney test when a normality test failed. NS, not significant. (**f**) Immunoprecipitation of Gpsm2 together with whirlin using anti-Gpsm2 serum. Membranes were immunoblotted with the antibodies indicated on the left. The experiment was replicated twice. (**g**) DIV3 hippocampal neurons electroporated with eGFP–myosin 15, DsRed-whirlin and myc-Gpsm2 show enrichment of all three proteins at the tips of filopodia (arrowheads). Filopodia are outlined with dotted lines. The LUT was modified (left, Orange hot) to better visualise the accumulation. *n*=5 independent experiments. Scale bar, 4 μm. (**h**) Actin assay shows that the combination of Gpsm2^FL^ and Gα_i3_ expression shift the F/G-actin ratio, whereas the Gpsm2^R318RfsX8^ mutation decreases this shift. Dot plot from five biological repeats (black bar represent mean values). **P<0.01 with Unpaired Student’s *t*-test, **P<0.01 with one sample *t*-test. (**i**) Actin assays on cultures of neurons show a shift in the F/G-actin ratio, suggesting a decrease in actin polymerization in the *Gpsm2* cKOs. Dot plot from five biological repeats (black bar represent mean values). **P<0.01 with one sample *t*-test.

To evaluate whether Gpsm2 regulates actin cytoskeleton dynamics we monitored the behaviour of individual actin-mEOS2 molecules using Single Particle Tracking combined with Photo-Activation Localization Microscopy (sptPALM) under Total Internal Reflection Fluorescence (TIRF) illumination, in the peripheral region of the growth cones (see Methods, Fig. 8a,b). We recorded trajectories longer than seven frames (median of nine frames, Fig. 8c), and fit the mean squared displacement (MSD) using a power function of time with exponent *α* (values between 0 and 2). This parameter reflects the curvature of the MSD function and the type of movement of actin-mEOS2 molecules, with *α* values close to 2 representing more directed trajectories, and *α* values close to 0 representing more static molecules (illustrated Fig. 8d). Comparisons analysis of the distribution of exponent alpha for control and *Gpsm2* cKO shows statistical differences in the most extreme *α* values (below 0.6 and above 1.4; Fig. 8e). This demonstrates that actin-mEOS2 exhibit different dynamical behaviour in the peripheral region of growth cones of control and *Gpsm2* cKOs. These results, together with the reduced neuronal outgrowth in *Gpsm2* cKO, suggest that Gpsm2 affects motility of the growth cone through a modulation of actin dynamics.

The retrograde flow of actin is a complex phenomenon that is the result of many individual mechanisms, including actin filament nucleation and polymerization, capping and depolymerization, in addition to mechanical forces experienced from myosin contractility and coupling to adhesion molecules. Although we do not know the molecular complex that could drive Gpsm2 control of growth cone motility, we were able to co-immunoprecipitate (co-IP) endogenous whirlin with Gpsm2 from hippocampi lysates (Fig. 8f). We also found that co-expression of myc-Gpsm2 with DsRed-whirlin and GFP–myosin 15 in young (DIV3) hippocampal neurons lead to a striking colocalization of the three proteins at the tips of filopodia, supporting a role for these proteins in young neurons motility (Fig. 8g). To test if Gpsm2 could modulate actin polymerization, we compared the ratio of F-actin with G-actin (F/G) in cells transfected with both *Gpsm2* and *Gnai3* constructs. When co-expressed, the proteins significantly increased the F/G-actin ratio by 138% (2.4±0.2) compared with the control, demonstrating its impact on actin polymerization (Fig. 8h). The Gpsm2^R318RfsX8^ variant of the protein resulted in a statistically significant reduction of this activation (1.6±0.1). A similar assay performed on cultured cortical neurons (see Methods), resulted in a 32% decrease in F/G-actin ratio in *Gpsm2* cKO neurons compared with controls (0.68±0.05) (Fig. 8i). These data support the hypothesis that Gpsm2 (with Gα_i3_) stimulates actin dynamics in neurons, and that some of the brain anomalies observed in patients, notably CC hypoplasia, could be related to a disruption of Gpsm2-dependant actin-based mechanisms.

## Discussion

In this study we show that the Gpsm2/Gα_i3_ module regulates actin polymerization during stereocilia elongation, and that a pathogenic mutation of either gene leads to abnormally short stereocilia, the likely cause of hearing loss in CMCS patients. We demonstrate that this function is due to a newly identified interaction between Gpsm2 and whirlin, a member of the stereocilia tip complex. Also, we show that a *Gpsm2* mutation affects CC formation and modulates neuronal outgrowth via the regulation of actin dynamics, supporting a global role for Gpsm2 in controlling the actin cytoskeleton.

**Figure 9:**
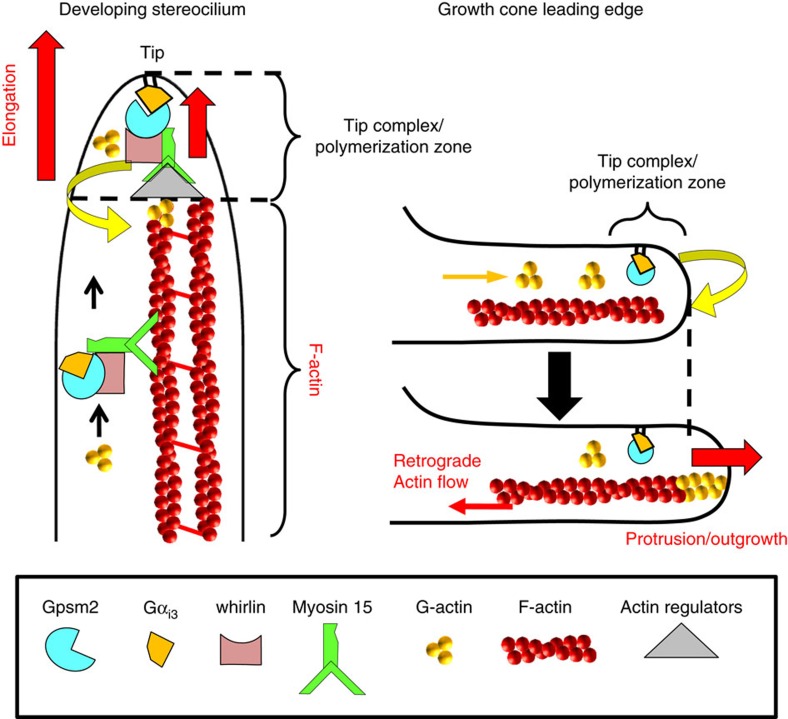
*Gpsm2* mutations affect stereocilia elongation and neuronal outgrowth by regulating actin dynamics at tip complexes. Mechanistic model for Gpsm2-dependent stereocilia elongation and neuronal outgrowth. Gpsm2 accumulates at the tip complex of both structures via the myosin 15 motor protein in stereocilia and a comparable motor protein in filopodia. Gpsm2-dependent macromolecular protein complexes modulate actin dynamics at the tip of stereocilia (growing end) or the leading edge of the growth cone, participating respectively in the elongation and motility of the two structures. See text for details.

Myosin 15 is the molecular motor responsible for delivering whirlin and Eps8 to the tips of actin-rich stereocilia and this ternary complex is required for elongation of nascent stereocilia. Our results show that myosin 15 and whirlin are also required for trafficking Gpsm2 and Gα_i3_, two new members of the stereocilia tip complex, an electron-dense structure believed to contain proteins that regulate actin polymerization (Fig. 9). Accordingly, when myosin 15 is non-functional, Gpsm2 and Gα_i3_ (this study), as well as whirlin^17^ and Eps8 (refs 19, 22) are all absent from the stereocilia tips. On the other hand, when *Whrn*, *Gpsm2* or *Gnai3* are mutated, myosin 15 still accumulates at the tips of short abnormal stereocilia. The failure of *Whrn*, *Gpsm2* and *Gnai3* cKOs stereocilia to elongate despite the localization of myosin 15, suggests that these proteins assemble in a macromolecular complex (including Eps8) to regulate actin polymerization at the stereocilia tips. Interestingly, it was recently shown that the distribution of whirlin on the tallest row of stereocilia was dependent upon the isoform 2 of myosin 15 (short form)^38^. It is therefore probable that Gpsm2 and Gα_i3_ are part of a preferential complex with myosin 15 isoform 2 and whirlin.

Our data extend the complexity of the interactions occurring at stereocilia tips during differentiation of HCs and provide new perspectives for the molecular machinery controlling actin polymerization. This complexity is highlighted by the increasing number of proteins identified and accumulating at the tip complex in a domain of ∼200 nm or less during stereocilia elongation (Fig. 9). In many cellular contexts, Gpsm2 and Gα_i3_ are at the interface between actin and the membrane, acting as a link between the two^39^,^40^. In stereocilia, G*α*_i3-GDP_ could be tethered at the plasma membrane via its myristoyl and palmitylate moieties, and bound to the C-terminal GoLoco motifs of Gpsm2. This would leave the tetratricopeptide repeats and linker region of Gpsm2 free to bind to various proteins (such as whirlin) to anchor or stabilize them, that could in turn interact with actin or other actin-regulatory proteins^41^, including Eps8 (refs 19, 22).

Our results significantly extend a recent report from Tarchini *et al*.^9^, showing the localization of Gpsm2 (LGN) and Gα_i_ protein at the tips of P7 mouse HCs stereocilia, and reporting deafness and shortened stereocilia in a *Gpsm2* mutant similar to ours. Importantly, we show that although myosin 15 is not required for the localization of Gpsm2 to the apical membrane crescent reported previously^6^,^8^, it is critical for the targeting of Gpsm2/Gα_i3_ to the stereocilia tip. This demonstrates that Gpsm2 binds with different protein complexes to engage in distinct molecular mechanisms at these locations.

Based on our data, the early deafness observed in *Gpsm2* cKO would be a consequence of a lack of postnatal elongation of stereocilia in IHC, the auditory sensory cells that are responsible for signal transduction, and which receive the vast majority of afferent innervation. Our results also uncovered Gα_i3_ as a specific molecular partner for Gpsm2 during stereocilia elongation, notably within the basal cochlear region, and as a candidate gene for early-onset progressive hereditary hearing loss. Some of the differences in hearing loss (earlier and comprising the entire tonotopic axis in *Gpsm2* mutants) probably reflect compensatory mechanisms by other Gα_i_ isoforms^42^.

Our data support the hypothesis that a decrease in actin polymerization, maybe through a disruption of the actin retrograde flow, underlies the reduced motility of *Gpsm2* cKO neurons. In young postmitotic neurons there is an obvious molecular similarity between the mechanism controlling the elongation of the stereocilia, and those controlling the movements of the growth cone, including the existence of a filopodial tip complex^43^,^44^,^45^ (Fig. 9). In both cases, it is the dynamic insertion of globular actin (G-actin) at the extremity of the structure (stereocilium or filopodium and lamellipodium) that allows elongation. This could be due to a role of Gpsm2 modulating the stability of the tip complex components, as we suggest for stereocilia elongation; a hypothesis supported by the endogenous co-IP of the Gpsm2 and whirlin from young hippocampi. All of the proteins identified so far that participate in stereocilia elongation are also expressed in the brain of mammals, including Gpsm2, Gα_i3_, myosin 15, whirlin and Eps8 (refs 46, 47, 48, 49, 50) (this manuscript). Myosin 15 is related to unconventional myosin 10 (MYO10), a powerful inducer of filopodia formation and elongation in neurons and other cells. Myosin 10 is present at the filopodia tip in bright puncta and remains there as the filopodia extend and retract^51^,^52^,^53^. We find myosin 15 enriched in neuronal growth cones and most filopodia, and accumulating with whirlin and Gpsm2 at filopodia tips when co-transfected into hippocampal neurons. Myosin 15 could have overlapping functions with myosin 10 in neuronal protrusions, through a protein complex similar to the tip complex identified in the inner ear. But it is also possible that other neuron-specific binding partners for Gpsm2 participate in this process in neurons. Further studies are required to explore the exact molecular mechanism underlying this result.

Previous work with a *Gpsm2* mouse model resulting in a truncated protein lacking the C-terminus suggested that cortical malformations in CMCS are due to abnormally localized apical progenitors, with no impact on neuronal production or forebrain thickness^54^,^55^. The authors however did not report on the morphology of the CC. In our *Gpsm2* cKO, we confirmed an overall normal cortical development and thickness, but we observed a severe hypoplasia of the CC, which appears to phenocopy a short and thin CC reported in the Palestinian patient carrying the p.R127X mutation in *GPSM2* (refs 2, 3). Our results demonstrating reduced neuronal outgrowth in *Gpsm2* cKO neurons offer a mechanistic explanation for CC hypoplasia. The diverse anomalies observed in CMCS patients can be understood in the context of three cellular phenomena: (1) the production and stability of the predicted truncated proteins, (2) the loss of GoLoco domains that are required for interaction with Gα_i_, and (3) the loss or maintenance of domains that can impact on other binding partners of Gpsm2. This last point is illustrated here with results from the Gpsm2^G491GfsX6^ and Gpsm2^R318RfsX8^ variants whose expression leads to the production of truncated proteins with an almost complete N-terminus that is still able to bind to whirlin and affect the ability of myosin 15/whirlin to generate filopodia. Notably, compared with the shortest Gpsm2^R127X^ variant^2^, the Gpsm2^R318RfsX8^ variant is associated with some of the most severe brain anomalies described so far, perhaps because the level of this truncated protein is barely detectable.

Recently, we showed that Gpsm2/Gα_i3_ regulates early planar cell polarity in inner ear epithelia by modulating tubulin dynamics in postmitotic cells in a mechanism reminiscent of those controlling oriented cell division^6^. Here, we show that the same complex controls actin dynamics not only in postmitotic HCs but also in neurons, through different binding partners. Because Gpsm2 has many interacting partners and is involved in microtubule and actin dynamics, in both dividing and postmitotic cells, each mutation identified in CMCS patients might affect a variety of mechanisms. The expression of Gpsm2 in both neurons and glia^44^ adds another level of complexity to this, as the role of the protein in the latter has not been assessed. On the other hand, in the inner ear the deficits are very similar regardless of the mutation, with early-onset deafness identified in all patients, highlighting the absolute necessity of an intact Gpsm2 protein for hearing.

Altogether our study strongly suggests that the aetiology of CMCS, notably its complexity and multi-syndromic aspect, is due to the multifunctional role of the Gpsm2/Gα_i3_ module on actin and tubulin dynamics, in proliferative and postmitotic cells. This new molecular role for Gpsm2/Gα_i3_ in the regulation of actin dynamics in epithelial and neuronal tissues show that this protein complex plays sequential and/or partially overlapping roles in mechanisms controlling the polarized growth of tissues. Taken together, our work emphasizes the importance of identifying all interacting partners of Gpsm2 and Gα_i3_ and the mechanisms associated with each interaction, in different (patho)physiological contexts.

## Methods

### Transgenic mice used in this study

All procedures involving animals were done in accordance to the European Communities Council Directives (2010/63/EU) and the French National Committee (2013-118) recommendations. The French ‘Ministere de l’Education Nationale, de l’Enseignement Superieur et de la Recherche’ approved all experiments under the authorisation no. APAFIS#1360-201508031720985 after agreement from the ethical committee of the University of Bordeaux. *Gpsm2/mPins* cKO was described previously^6^. Generation of conditional *Gnai3* KO mice. Exon 6 of the *Gnai3* gene was flanked by loxP-sequences, because in the global knockout mouse this exon was successfully deleted^56^. For the positive selection of ES cell clones, a loxP-flanked *TK-Neo* resistance cassette was inserted in reverse orientation in intron 6 of the *Gnai3* gene at the 3′ site. For negative selection a DTA cassette was inserted after exon 7. Subsequently, the loxP-flanked *TK-Neo* resistance cassette was deleted *in vitro*. Correctly targeted ES cell clones were identified and used for generation of chimeric mice, that is, SV129/C57BL/6 genetic background. After germ line transmission the mutant mice were backcrossed onto a C57BL/6 genetic background. In this study, *Gnai3* cKO were achieved by crossing Gα_i3_^fl/fl^ and *Foxg1-Cre* mice. S*haker 2* (*Myo15* allele) and *whirler* (*Whrn* allele) mutant mice were maintained at the NIDCD and all procedures were approved by the Animal Care and Use Committee (ACUC #1263-16). Foxg1^tm1(cre)Skm^ (*Foxg1*-Cre) and Emx1^B6.129S2-Emx1tm1(cre)Krj/J^ (*Emx1*-Cre) stocks were obtained from the Jackson Laboratory (Bar Harbor, ME), Tg^(Pou4f3-cre)1Devet^ mice was a gift from Douglas Vetter (Tufts University, Boston, MA, USA), B6.Cg-Gt(Rosa)26Sortm6(CAG-ZsGreen1)Hze/J (Ai6) mice were purchased from The Jackson Laboratory. All *Gpsm2* conditional knockout lines (cKO) were generated by crossing *Gpsm2* flox/flox animals^6^, whereas the *Gnai3* conditional knockout (*Gnai3* cKO) line was generated by crossing *Gnai3* flox/flox animals. The resulting *Gpsm2* cKO still expresses a short 147 amino-acid protein corresponding to the N-terminal of the Gpsm2^FL^, whereas the *Gnai3* cKO is a full conditional knockout. The recombination pattern in Foxg1-Cre mice closely matches the expression pattern of endogenous Foxg1. By E9.5/10.5 the expression of Cre is strongly driven in both the telencephalon and the entire otic vesicle/otocyst (early inner ear) of embryonic mice. Recombination occurs efficiently in both HCs and supporting cells in the cochlea (and vestibular system), and in most neurons of the telencephalon (glutamatergic and gabaergic), notably in the cortex and hippocampus^57^,^58^. Pou4f3 is expressed only in postmitotic HCs^30^, and Emx1 is expressed in the entire telencephalon as early at E10.5 (ref. 59). The Cre reporter mouse strain B6.Cg-Gt(Rosa)26Sortm6(CAG-ZsGreen1)Hze/J (Ai6), which induces the expression of ZsGreen from the Rosa26 locus, was used for evaluation of *Pou4f3*-Cre-induced recombination localization. Sprague Dawley Rats were obtained from Janvier (France).

### Cochlear explants cultures and PTX treatment

For cochlear cultures, cochleae from P0/P1 rat are placed in culture and the next morning (12–16 h later) PTX was applied at 1– 100 ng ml^−1^, using a stock solution (50 μg ml^−1^, Sigma) as reported previously described^6^. Half of the culture medium was changed every 2 days, and after 8 DIV the tissue were fixed before labelling with phalloidin fluorescein isothiocyanate (Sigma Aldrich) or processed for SEM.

### Cochleae immunostaining

Inner ears from mice or rats were harvested at specific time points between P5 and P21. Up to P5 mice and rats were killed by decapitation, and in later stages, the animals were anaesthetized by CO_2_ before decapitation. The inner ears were dissected and fixed in 4% paraformaldehyde for 1.5– 24 h at 4 °C for immunostaining. Fixed and dissected cochlear duct were processed for immunocytochemistry as previously described^6^. Some cochleae were mechanically scratched using forceps tips within the mounting medium to isolate stereocilia. The primary antibodies were as follow: anti-Gα_i3_ (ref. 60 or Sigma G4040, 1:600), custom-made anti-Gpsm2 (ref. 6, pAb 1:500), anti-myosin 15 (PB48, ref. 50, pAb, 1:400), anti-whirlin (HL5136, ref. 17, pAb, 1:200), or anti-Eps8 (BD Transduction Laboratories, mAb, #610143, 1:200). Samples were incubated with primary antibodies for 1.5/2 h at room temperature (RT) to overnight (ON) at 4 °C with the primary antibodies, washed and secondary antibodies applied (anti-mouse or anti-rabbit conjugated with Alexa-488, -594 or ATTO647N (Life Technologies) for 1 h at RT. Phalloidin conjugated with fluorescein isothiocyanate (P5282 Sigma, 1:500) or TRITC (P1951 Sigma, 1:500) were added for 1 h at RT to label F-actin and thereby stereociliary hair bundles of HCs. When comparing staining between controls and knockout, the two cochleae were processed in the same tube at P5. For later stages, they were processed in different tubes as only a small piece can be dissected at this stage, which renders final identification difficult. The samples were placed in mounting medium (Prolong Gold antifade reagent, Life Technologies) and flattened with a glass coverslip under a microscope.

For image acquisition, we used a confocal/STED microscope (TCS SP8; Leica) with a module STED × 3. Imaging was done using a Z step from 0.25 to 0.35 μm. STED microscopy were performed with an objective 100 × 1.4 numerical aperture oil immersion STED objective. We used Atto 488 Phalloidin (Sigma, #49409, 1:600) with a depletion laser of 592 nm, goat anti-mouse Atto 647 N (Sigma, #50185-1, 1:300) and goat anti-rabbit Alexa Fluor 594 with a depletion laser of 775 nm. Confocal images were processed in Volocity software (Perkin Elmer) and Adobe Photoshop or ImageJ.

### SEM and measurements

The inner ear (cochlear and vestibular system) of mice aged P5 and P21 were harvested and immersed in 2.5% glutaraldehyde in 0.1 M cacodylate buffer, pH 7.35, with 3 mM CaCl2 for 24 h or more. The tissues were postfixed in 1% OsO4 in the same buffer, dissected and double processed with thiocarbohydrazide followed by OsO4 (ref. 61) before dehydration through an alcohol series and critical point drying with CO2. After mounting on specimen support stubs, samples were sputter coated with platinum. Samples were examined with a JEOL 6700 F cold field emission scanning electron microscope operating at 3 or 5 kV. Measurements were made on the longest row of stereocilia closest to the kinocilium on 84 control stereocilia/16 HCs/2 mice and 75/24/2 (for P5 and P21), on *Gpsm2* cKO 36/8/2 and 108/21/2 (For P5 and P21), and on *Gnai3* cKO 95/18/2 and 60/14/2 (For P5 and P21) in at least four different cochleae. The quantification of the number of stereocilia were made on 23 control HCs/2 mice and 12/2 (for P5 and P21), on *Gpsm2* cKO 16/2 and 23/2 (for P5 and P21) and on *Gnai3* cKO 19/2 and 32/2 (for P5 and P21) in at least four different cochleae.

Images were collected from the basal or middle turn of the cochlea, defined as ∼20/30% and 50/60% of the total length of the organ of Corti from the base. At each location, the hair bundles were viewed both from behind the longest row of stereocilia to view the height of the hair bundle as well as approximately perpendicular to the apical surface of the HC or toward the inner aspect of the bundle to examine its overall morphology and composition. To estimate bundle height, measurements were made from images at calibrated instrument magnifications of × 20,000 or occasionally × 10,000. Although we might have underestimated the actual stereocilia length, all efforts were taken to minimize the effect of parallax. Measurements were taken from bundles viewed from the lateral side toward the medial side (from the stria toward the modiolus), so that the row of longest stereocilia were imaged from the ‘rear.’ Samples were tilted and rotated so that the row of longest stereocilia was approximately perpendicular to the direction of view. Stereo-imaging was used in a few cases to gain an indication of possible errors in length measurements from 2D images. From the anaglyphs generated by a pair of images separated by 8 degrees of tilt, height measurements were obtained using analySIS software. These revealed little difference in the height measurement from that obtained from the 2D image of a stereocilium at close to perpendicular to its long axis. Measurements were made using ImageJ software.

### ABR and vestibular tests

ABR is measured by averaging the evoked electrical response recorded via subcutaneous electrodes. ABR to click and pure tone stimuli were recorded in anaesthetized mice aged 6 weeks. All physiological recordings were performed under anaesthesia (75 mg kg^−1^ ketamin hydrochloride, Ketavet, Pharmacia, Pfitzer, Karlsruhe Germany, 5 mg kg^−1^ xylazin hydrochloride (Rompun 2%, Bayer Leverkusen, Germany), 0.2 mg kg^−1^ atropine (Atropinsulfat B Braun, Melsungen, Germany) in a soundproof chamber (IAC, Niederkrüchten, Germany). In short, ABR thresholds were determined with click (100 μs), and pure tone stimuli (2–45.3 kHz, 3 ms duration).

For vestibular test, *Gpsm2 and Gnai3* cKO mice and their control littermates (6 weeks old) were housed in a controlled environment (20–23 °C) with free access to food and water and maintained on a 12 h/12 h day/night cycle, light on at 7 am. Mice littermates were housed in collective cage and behavioural experiments were performed between 1 and 5 pm. Open-field and Forced swim test activities were analysed with Ethovision (Version 11.5, Noldus Technology, Wageningen, The Netherlands). In the open-field test, mice were placed in the arena (30 × 40 cm) and activity was recorded for a period of 10 min. The total distance travelled and the number of rotations were measured (every 360° turn is counted as one rotation). In the forced swim test, mice were placed in a cylinder (height, 30 cm; diameter, 21 cm) filled with water (25 °C). The activity, the total immobility and the number of rotation was recorded for a 2 min period. Immobility is defined by movements inferior to 2 cm s^−1^.

### Filopododia quantifications in COS-7 cells

The constructs used were as follows: untagged Gα_i3_ (Origene), pK-myc-Gpsm2^FL,48^, pEGFP–c-MyoXVa, pDsRed-c-whirlin and DsRed-empty (Clontech) or pK-myc-empty. Site-directed mutagenesis was used to generate mutations R127X (Gpsm2^R127X^), Q562X (Gpsm2^Q562X^), G491GfsX6 (Gpsm2^p.G491GfsX6^) and R318RfsX8 (Gpsm2^R318RfsX8^) on Gpsm2 (QuickChange, Stratagene). We used an anti-myc (Covance mAb, #MMS-150P-200, 1:1,000), anti-Gα_i3_ (ref. 60 and Sigma G4040, 1:600), anti-GFP chicken (Abcam, pAb #ab13970, 1:3,000), anti-DsRed (Living color, mAb #632392, 1:1500 and Living color, pAb #632496, 1:2,000), and Phalloidin conjugated with coumarin (Sigma #P2494, 1:500). Images were acquired using Zeiss Axovision 4.7 and processed through Photoshop. For long-term assays (48 h), we considered a COS-7 (ATCC-American Type Culture Collection) ‘filopodial cell’ a cell with at least five long filopodia. For short-term assays of filopodia length, we split the cells after 48 h of transfection, and re-plated them on coverslips previously coated with 5 μg ml^−1^ of poly-L-Lysine (PLL), and let them adhere for 2 h, before they are processed for immunocytochemistry. For the quantification of percentage of colocalization at the tip of filopodia, 500,000 COS-7 cells were transfected by nucleofection using an Amaxa nucleofector kit (Lonza). After, 48 h the cells were re-plated on coverslips previously coated with 5 μg ml^−1^ of PLL, and processed for immunocytochemistry after a 2 h adhesion. Cell appearing unhealthy were excluded from the analysis. Quantifications were performed blind to the experimental group using ImageJ.

### Co-IP

For Co-IP, HEK293T (ATCC-American Type Culture Collection) were cultured on 10 cm dishes, transfected using polyethylenimine and harvested after 48 h in cold PBS. We used myc-tagged Gpsm2 constructs and/or GFP–whirlin (whirlin was subcloned into the mammalian expression vectors pEGFP–C1-Clontech) or untagged whirlin constructs (generous gift from Professor C Petit, Pasteur Institute, Paris) and pEGFP–C3. Extracts were solubilized with Triton-X-100 and a cocktail of protease inhibitor (Complete ethylenediaminetetraacetic acid (EDTA)-free, Roche), and processed as previously described^48^. In brief, lysates were incubated with protein A/G or A resin ON with primary antibodies. The antibodies used were anti-myc (Millipore, pAb #06-549, 10 μg ml^−1^), anti-GFP (Millipore, pAb #Ab3080P, 10 μg ml^−1^), and IgG from rabbit serum (Sigma, pAb #I5391, 3 μg ml^−1^). After extensive washes, the beads were re-suspended in sodium dodecyl sulfate (SDS) sample buffer immunoprecipitated proteins were analysed by SDS–PAGE and immunoblotting. Proteins were visualized using chemiluminescence-based immunodetection of horseradish peroxidase (Amersham). Each co-IP was replicated at least three times.

For endogenous co-IP, 10 hippocampi from P21 male and female rats were harvested and solubilized with 1% DOC and 1% Triton-X-100 in 50 mM Tris buffer pH 8 with protease inhibitors, and processed as previously described^48^. In brief, lysates were incubated with 20 μl of pre-immune or Gpsm2 serum^48^ and with protein A resin ON. For immunoblots, we used anti-whirlin (ref. 17, pAb 10 μg), and the anti-Gpsm2^Linker^ (generous gift from Joe Blumer, Medical University South Carolina, USA) (ref. 46, pAb, 1:1,000).

### GST pull-down

Different whirlin regions were cloned by polymerase chain reaction and inserted into pGEX-4T-1 (GE) to create in-frame fusions with GST. GST-tagged whirlin 1–229 containing PDZ1, GST-whirlin 1–362 containing PDZ1 and PDZ2, GST-whirlin 276–362 containing PDZ2, GST-whirlin 276–907 containing secondary structures between PDZ2 and PDZ3, GST-whirlin 276–672 containing PDZ2 and the HN-L2 region, GST-whirlin 673–907 containing the proline-rich region and PDZ3, GST-whirlin 810–907 containing only the PDZ3 and GST-whirlin 363–809 containing the HN-L2 and proline-rich region were purified from *Escherichia coli* strain BL21 supernatants by standard affinity purification on glutathione-Sepharose 4B beads. In brief, bacterial pellets were re-suspended and incubated for 30 min on ice in TBS pH 7.5 lysis buffer containing 150 mM NaCl, 10–20 mM phosphate buffer and 0.1 mg ml^−1^ lysozyme. Protease inhibitors were then added along with DTT (15 mM), EDTA (10 mM) and sarkosyl to a final concentration of 1.5%. The mixture was gently shaken, incubated for 15 min on ice and centrifuged for 1 h at 186,000 × *g*. sarkosyl in the supernatant was neutralized with 2% Triton X-100 and the supernatant was incubated for 3 h at 4 °C on glutathione-sepharose 4B beads. The beads were washed three times with TBS pH 7.5 containing 0.1% Triton X-100 and re-suspended in TBS pH 7.5 with proteases inhibitors for later use.

A pull-down assay was performed with myc-Gpsm2^R318RfsX8^ overexpressed in HEK293T. HEK293T are re-suspended and sonicated in Tris-HCl pH 7.5 containing 5 mM EDTA, 1 mM sodium fluoride, 1 mM sodium orthovanadate, proteases inhibitors and then solubilized 30 min with 1% Triton X-100 and 0.5%SDS. The lysate was centrifuged for 30 min at 150,000 × *g*. The supernatant containing myc-Gpsm2^R318RfsX8^ was incubated ON at 4 °C with GST alone or GST-whirlin fusion proteins. After four washes with buffer containing 0.1% Triton X-100, the bead pellets were re-suspended in SDS sample buffer and subjected to SDS–PAGE and immunoblotting. Interaction was evaluated with anti-myc antibody (Covance, mAb, 1:1,000). To evaluate GST amounts, the samples are loaded on SDS–PAGE and stained with Coomassie blue.

### Western blot and immunoblot

HEK293T cells in six-well plates were co-transfected with 50, 100, 200 and 300 ng of myc-Gpsm2^FL^ in the presence of a constant concentration of DsRed-whirlin or DsRed-empty vector (50 ng), or with stable levels of Gpsm2 with increasing concentrations of whirlin. In each condition, the total plasmid concentration was balanced with a control plasmid to a total per dish of 350 ng plasmid. After 48 h, the cells were collected using cold PBS, centrifuged and solubilized by sonication. Lysates were re-suspended in SDS sample buffer and subjected to SDS–PAGE and immunoblotting with anti-myc (Covance, mAb 9E10, 1:1,000), anti-whirlin (Santa Cruz Biotech, mAb #sc-365250, 1:1,000), anti-DsRed (Living color, pAb #632436, 1:1,000), anti-GAPDH (Millipore, mAb #MAB374, 1:1,000), anti-GFP (Living Color, mAb #632381, 1:1,000) followed by secondary antibodies (donkey anti-rabbit #CNA394V 1:5,000 or sheep anti-mouse IgG #CNA931V 1:5,000 conjugated to horseradish peroxidase, GE Healthcare UK). The membranes were processed with chemiluminescence (ECL, Thermo Scientific) as previously described^6^.

To evaluate the level of expression of Gpsm2 truncated forms, we transfected the variants of Gpsm2 in HEK293T and after 48 h the cells were harvested and the amount of protein evaluated by BCA assay. For each condition we pooled two wells (six-well plates), and equivalent protein levels were loaded and separated on polyacrylamide gels, and immunoblot performed with anti-myc antibody (Covance), and anti-GAPDH (Millipore). Quantifications were made using GS-800 calibrated densitometer Bio-Rad and Quantity One Analysis software (Bio-Rad). Unprocessed original scans of blots are shown in Supplementary Figs 3–7.

### Actin polymerization assays

The amount of F-actin and G-actin was evaluated according to the Cytoskeleton Actin Polymerization Assay Kit (BK037, Cytoskeleton) protocol and as previously described^62^. HEK293T were transfected with a combination of pK-RFP/pK-YFP (control), or myc-Gpsm2/YFP-Gα_i3_ (ref. 48). 24 h after HEK293T transfection we reduced the levels of fetal bovine serum in the medium from 10 to 1%, and the next day, the cells were stimulated with fresh medium containing 10% fetal bovine serum for 30 min, then re-suspended in F-actin stabilization buffer with adenosine triphosphate (1 mM) and protease inhibitors.

Cortical and hippocampal neurons from P0 controls and *Gpsm2* cKO*s* were dissociated and plated at a density of 200,000 cells per dish, and stimulated with KCl before harvesting at DIV3. For neuron stimulation, we used 20 mM KCl at 37 °C for 1 min, then collected the neurons in cold PBS before pelleted them at 3,000 × *g* for 5 min at 4 °C, and stored at −80 °C. To separate F-actin (pellet) from G-actin (supernatant) a 1 h centrifugation at 100,000 × *g* was performed on the lysate, then the pellet was re-suspended in a volume equivalent to the supernatant using F-actin depolymerizing buffer. Actin levels were quantified by immunoblot using an anti-actin antibody (Cytoskeleton, #AANO1, 1:500). For each condition we pooled three petri dishes. The expression levels were determined using a GS-800 calibrated densitometer and Quantity One Analysis software (Bio-Rad), and represented as a percentage of control band intensity. We used a total of 14 controls and 10 *Gpsm2* cKO*s* mice.

### Histology

For histology, brains were harvested and fixed in Bouin’s fixative (Electron Microscopy Sciences) ON, dehydrated in ethanol, paraffin-embedded, and coronal sections (20 μm) obtained, before being stained with hematoxylin and mounted with Entellan (Millipore). Brain sections were examined using Leica MZ-16 stereomicroscope using the NanoZoomer 2.0-HT slide scanner and analysed with the Hamamatsu NDP viewer software (Hamamatsu).

### Neuronal cultures and transfections

Hippocampal neurons were dissociated from E 18 rat embryos as described^48^ and electroporated with 2 μg of eGFP–Myo15a, myc-Gpsm2^FL^ and DsRed-whirlin using Amaxa nucleofector kit (Lonza). Approximately 500,000 cells were transfected via nucleofection plated on coverslips treated with 10 μg ml^−1^ of PLL. After 2 days, neurons were fixed for 10 min with 4% paraformaldehyde at RT, then pre-incubated 30 min at RT in permeabilization buffer (PBS, 10% NGS, 0.1% triton). Cells were then incubated at RT for 1 h with chicken anti-GFP (Abcam, pAb #ab13970, 1:3,000), anti-myc (mAb, Covance, 1:1,000) and anti-DsRed (Clontech, pAb #632496, 1:3,000). Fluorescent images of the neurons were obtained using a confocal microscope (Leica SP8) and processed with Adobe Photoshop.

### Videomicroscopy and sptPALM-TIRF

Regular 18-mm glass coverslips were incubated for 2 h at 37 °C with 1 mg ml^−1^ PLL in 0.1 M borate buffer (pH 8.5), rinsed with H_2_O, then incubated 2 h at 37 °C with 4 μg per coverslip of goat anti-human Fc (Jackson Immunoresearch) in 0.2 M boric acid (pH 8.5), before another incubation ON at 4C with 0.2 μg per cover (stpPALM-TIRF) and 0.6 μg per coverslip (time-lapse) of N-cadherin-Fc. Before use, the coverslips were rinsed again with boric acid. For laminin, coverslips were coated with 1 mg ml^−1^ PLL and then laminin (5 μg per cover).

For time-lapse experiments (growth cone outgrowth), hippocampal neurons from newborn (P0/1) pups were plated at a density of 50,000 cells per coverslip. Neurons were used after 2 DIV. Neurons were covered with 700 μl of Tyrode solution (120 NaCl mM, 5 KCl mM, 2 MgCl2 mM, 2 CaCl2 mM, 25 mM 4-(2-hydroxyethyl)-1-piperazineethanesulfonic acid, and 30 mM d-glucose, pH 7.4), and observed under an inverted Leica DMI 6000 microscope (Leica Microsystems, Wetzlar, Germany) using a HCX PL APO CS × 63 oil 1.32 numerical aperture objective and differential interference contrast illumination (Lumencor, Beaverton, USA). Images were acquired every 1 min for 30 min using a HQ2 camera (Photometrics, Tucson, USA) driven by the MetaMorph software (Molecular Devices, Sunnyvale, USA). The multi-positions were done with a motorized stage Scan IM (Märzhäuser, Wetzlar, Germany). Temperature was maintained at 37 °C. The system was controlled by MetaMorph software. Quantification of neuron growth cone speed was performed using ImageJ software plugin ‘manual tracking’. The average speed (in μm min^−1^) was quantified as this distance divided by 30 min. A ‘pause’ is when no movement (within the pointing accuracy of the growth cone centroid) of the growth cone is observed over two consecutive time-lapse images (1 min image^−1^), while maintaining filopodial activity.

The stpPALM-TIRF experiments were done as described in ref. 63. In brief, coverslips were mounted in a chamber and placed on a Nikon Ti Eclipse inverted microscope (Nikon France S.A.S., Champigny-sur-Marne, France) equipped with a TIRF arm coupled to a fibre optic linked to a four-colour laser bench (Roper Scientific). Images were acquired using an Apo TIRF 100 × oil numerical aperture 1.49 objective, and a sensitive EMCCD camera (Evolve, Photometrics, Tucson, USA). Photoswitching of mEos2 was done at 405 nm, converting the molecule into a red-emitting form, which was detected using the 561 nm laser and a long-pass emission filter. All the equipment was driven by the software Metamorph. Image stacks of 320 frames were acquired in time-lapse mode at a frequency of two images per second, with a camera exposure time of 250 ms. This procedure ensures that fast-diffusing actin monomers which contribute to a blur in the images are eliminated from subsequent analysis, and that only slowly moving actin molecules incorporated in filaments are retained^63^,^64^. Experiments were done on 11 growth cones for control and 12 growth cones for cKO Gpsm2 neurons, from at least three separate experiments.

### Speed and trajectories analysis

Actin trajectories recorded by sptPALM were computed and analysed using custom-made algorithms written as a MetaMorph plug-in as described in ref. 63. Single-molecule localization was performed using a wavelet-based algorithm, and trajectories were computed using a simulated annealing algorithm^65^. The trajectory duration, which corresponds to the time during which single mEOS2 fluorophores emit red light upon 561 nm laser illumination, follows an exponential distribution strongly shifted to short values. Only trajectories longer than seven frames in regions of interest were kept, which yielded a median of around nine frames. The proportion of trajectories with more than seven points is 80% in control and 78% *Gpsm2* cKO growth cones respectively. The MSD function was computed for each trajectory over time, and fit by the power law MSD=4*Dt^α^* using Kaleidagraph 4.1, where *t* is the time, *D* is an adjustable coefficient and the exponent *α* (values between 0 and 2) reflects the curvature of the MSD function and the type of movement. For highly directed trajectories, the MSD is a quadratic function of time, thus *α* is close to 2 (ref. 64, 66). The numbers of trajectories analysed were 1344 (controls) and (1121) mutants.

Statistical analyses were carried out using Prism statistical package (GraphPad). Normality of distribution and homogeneity of variance were validated and statistical significance between means was calculated using unpaired Student’s *t*-test or Mann–Whitney test when normality test failed. *P*<0.05 was considered significant.

### Data availability

All data are available from the authors.

## Additional information

**How to cite this article:** Mauriac, S. A *et al*. Defective Gpsm2/Gα_i3_ signalling disrupts stereocilia development and growth cone actin dynamics in Chudley-McCullough syndrome. *Nat. Commun.* **8**, 14907 doi: 10.1038/ncomms14907 (2017).

**Publisher’s note**: Springer Nature remains neutral with regard to jurisdictional claims in published maps and institutional affiliations.

## Supplementary Material

Supplementary Information Supplementary Figures.

Supplementary Movie 1 Time-lapse of growth cones from control and mutant. Thirty minutes DIC time-lapse movie illustrating the displacement of a control followed by a *Gpsm2* cKO growth cone on N-cadherin substrate.
