# Author Correction: Defective Gpsm2/Gα_i3_ signalling disrupts stereocilia development and growth cone actin dynamics in Chudley-McCullough syndrome

**DOI:** 10.1038/ncomms16188

**Published:** 2018-05-25

**Authors:** Stephanie A. Mauriac, Yeri E. Hien, Jonathan E. Bird, Steve Dos-Santos Carvalho, Ronan Peyroutou, Sze Chim Lee, Maite M. Moreau, Jean-Michel Blanc, Aysegul Gezer, Chantal Medina, Olivier Thoumine, Sandra Beer-Hammer, Thomas B. Friedman, Lukas Rüttiger, Andrew Forge, Bernd Nürnberg, Nathalie Sans, Mireille Montcouquiol

Nature Communications 8: Article number: 14907; DOI: 10.1038/ncomms14907 (2017); Published: 04 07 2017; Updated: 05 25 2018

The original version of this Article contained an error in the spelling of the author Aysegul Gezer, which was incorrectly given as Aysegul Geyser. This has now been corrected in both the PDF and HTML versions of the Article.

