# Supplementary material for: Defective Gpsm2/Gαi3 signalling disrupts stereocilia development and growth cone actin dynamics in Chudley-McCullough syndrome
Source: Nat Commun. 2017 Apr 7;8:14907. doi: 10.1038/ncomms14907 (PMC5385604; doi:10.1038/ncomms14907)
Supplement: Supplementary Information — Supplementary Figures. [file ncomms14907-s1.pdf]

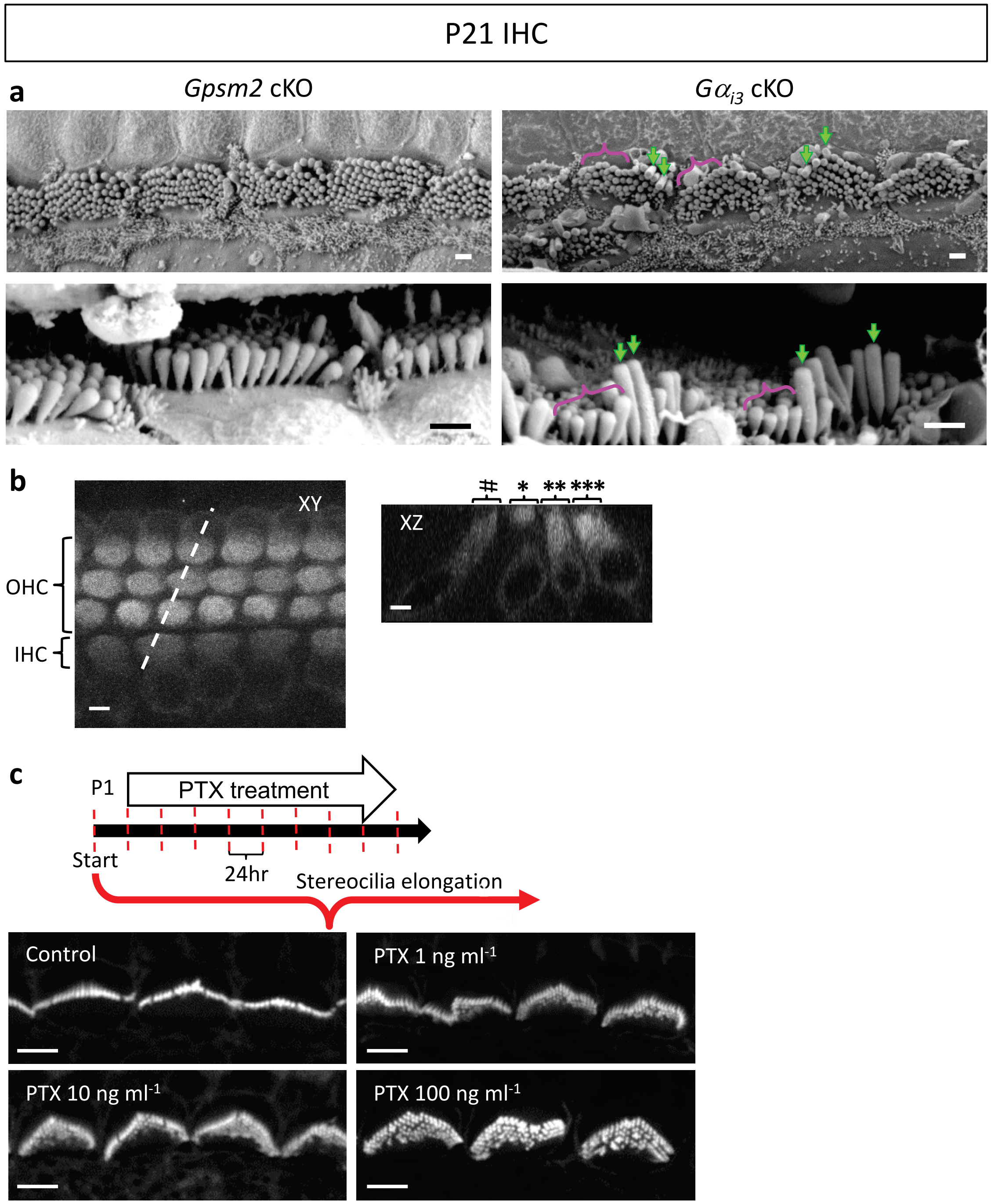

Supplementary Figure 1. *Gpsm2* and *Gnai3* mutations inhibit stereocilia elongation. (a) SEM of surface (top panels) and lateral (bottom panels) views of midbasal region of the cochlear epithelia of P21 mouse in *Gpsm2* (left panels) or *Gnai3* (right panels) cKOs. Whereas in *Gpsm2* cKO all of the stereocilia are shorter, in *Gnai3* cKO we observed short (magenta brackets) and long (green arrows) stereocilia within the same hair bundle. Scale bar, 1 µm. **(b)** Surface view (XY) and lateral view (XZ) of the cochlear epithelium of P5 pups (*Pou4f3*-Ai6) resulting from a cross between *Pou4f3*-Cre mice and Ai6 mice. ZsGreen is expressed in IHCs and OHCs, validating the Cre recombinase excision in these cells. **(c)** Timeline of PTX treatment for cochlear explants. The cochleae are harvested at P1 and after 24 hr *in vitro*, the PTX is applied for 8 additional days *in vitro* before immunocytochemistry or SEM treatment. Bottom panel: surface views of postnatal cochlear explants treated for 8 days with increasing concentrations of PTX. The hair bundles display a phenotype similar to *Gnai3* cKOs, with supernumerary rows of shorter stereocilia. Scale bar (b,c), 4 µm.

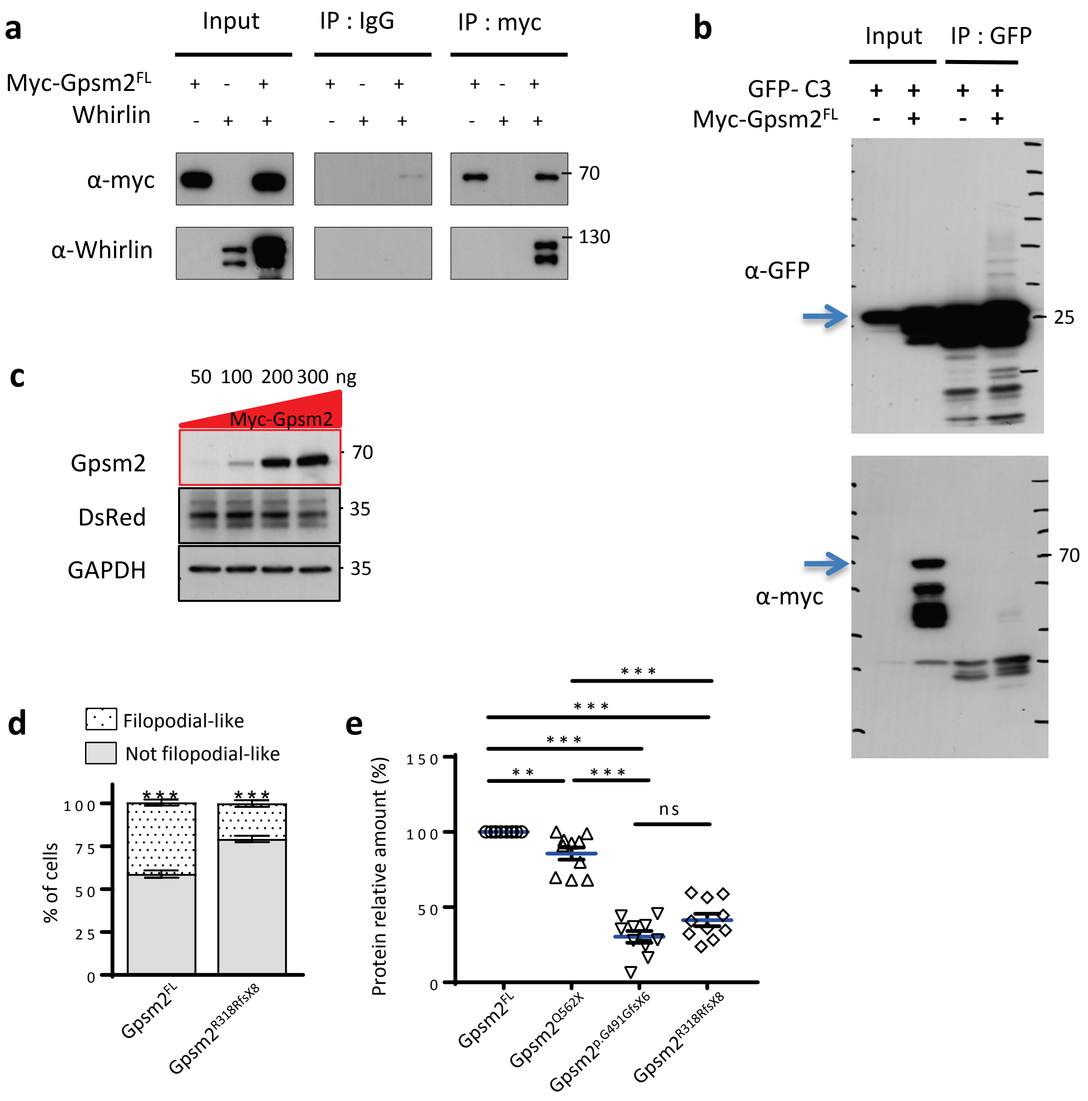

Supplementary Figure 2. A new complex between Gspm2 and whirlin controls filopodia elongation. (a) Immunoprecipitation of Gpsm2^{FL} and whirlin with anti-myc. Membranes were immunobloted with the antibodies indicated on the left. **(b)** Immunoprecipitation of Gpsm2^{FL} with GFP alone. **(c)** Increasing doses of Gpsm2 has no effect on an empty vector (DsRed) levels. **(d)** Expression of Gpsm2^{R318RfsX8} variant with whirlin and myosin 15 leads to a decrease in the number of COS-7 with filopodia when compared with cells transfected with myc-Gpsm2^{FL}. Data are represented as the percentage of the total number of the filopodia counted in each condition (n=154 to 173 cells) from three separate experiments. $***$ $P < 0.001$ with unpaired student's t test. (±s.e.m.). **(e)** Quantitative analysis of the various protein levels of myc-Gpsm2. $*$ $P < 0.01$, $***$ $P < 0.001$ with one sample t test or one way Anova (post-hoc Bonferonni's test). ns, not significant.

Blots for Fig. 6e

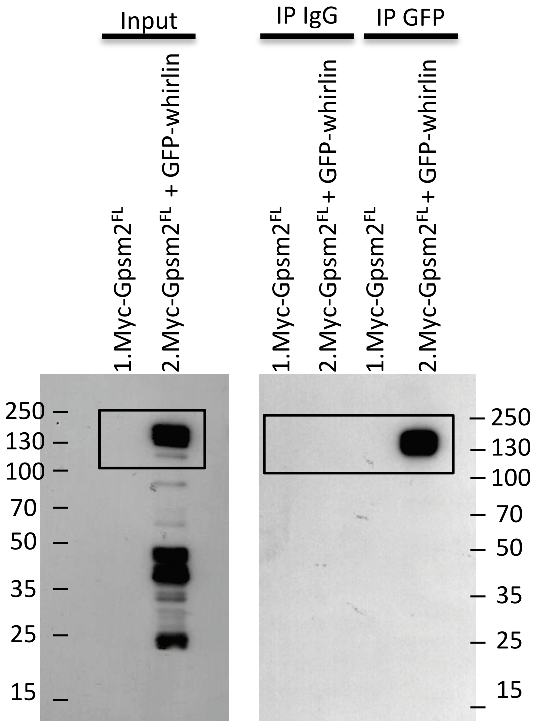

Anti-whirlin mAb G-8 (1:1000)
IP with anti-GFP pAb Millipore / IgG Rabbit serum

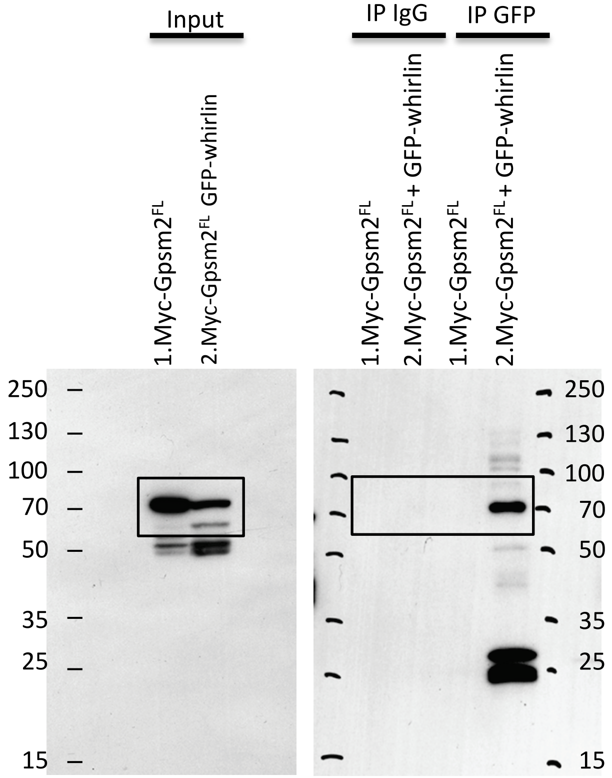

Anti-myc mAb 9E10 (1:1000)
IP with anti-GFP pAb Millipore / IgG Rabbit serum

Blots for Fig. 6f

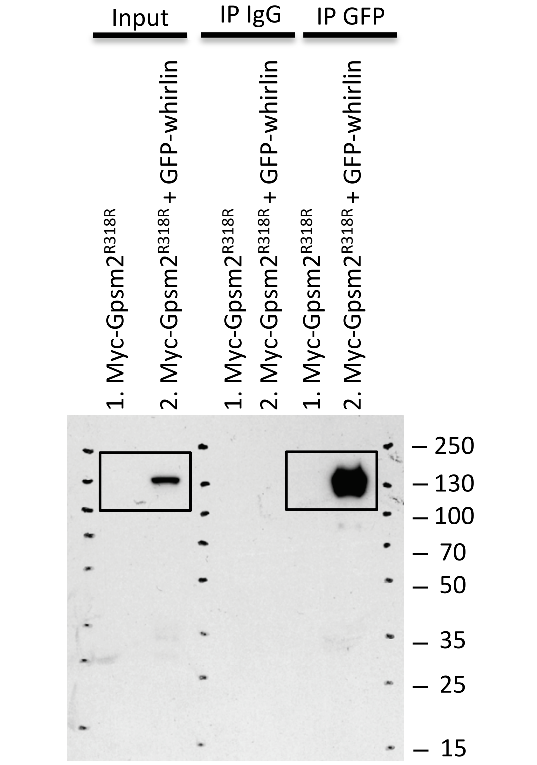

Anti-whirlin mAb G-8 (1:1000)
IP with anti-GFP pAb Millipore / IgG Rabbit serum

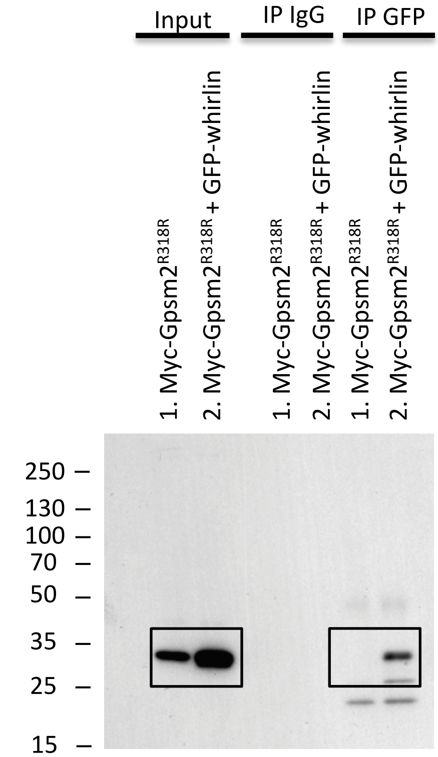

Anti-myc mAb 9E10 (1:1000)
IP with anti-GFP pAb Millipore / IgG Rabbit serum

Supplementary Figure 3. Full scans of Western blots shown in Figures 6e,f.

Blots for Fig. 6g

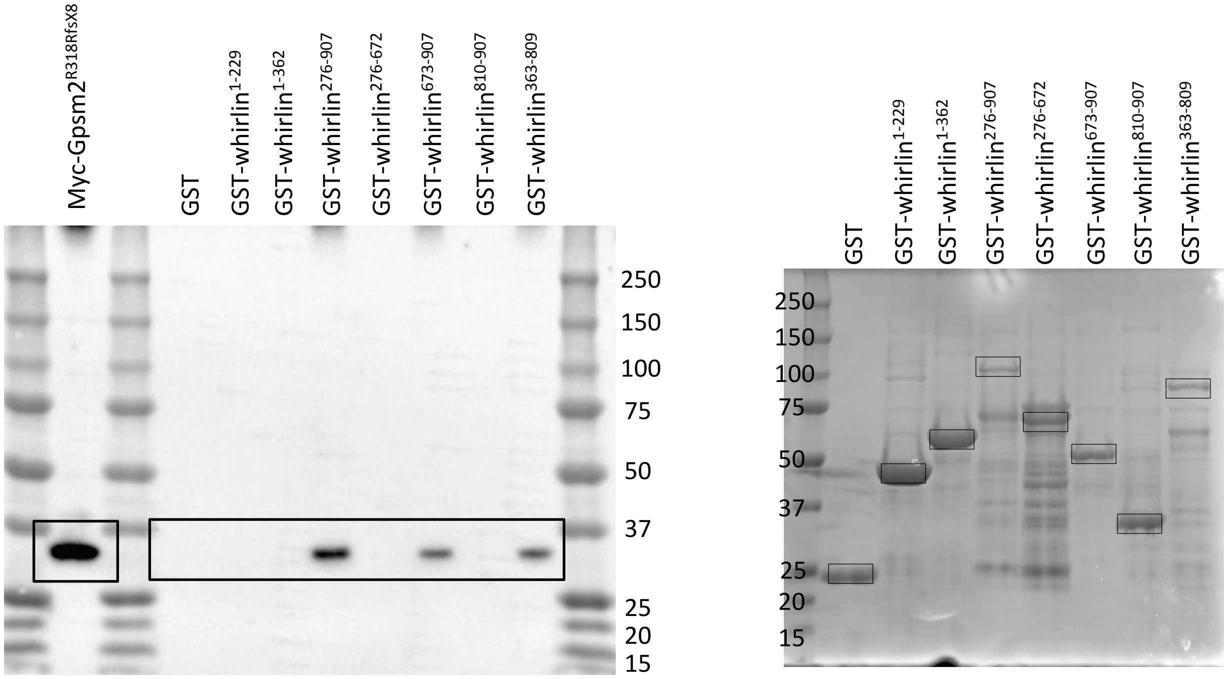

Anti-myc mAb 9E10 (1:1000)

Coomassie

Blots for Fig. 6h

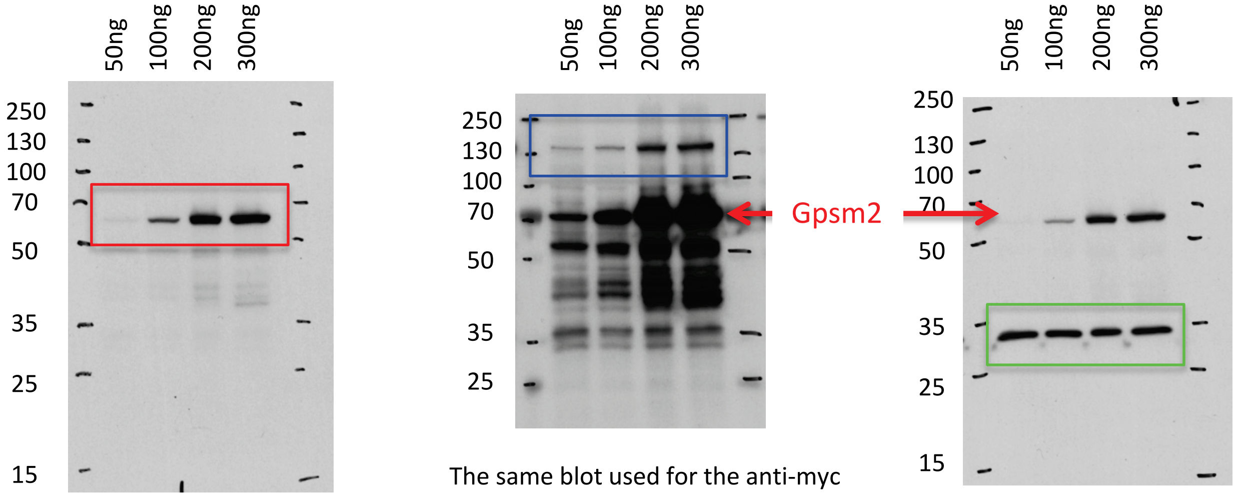

Anti-myc mAb 9E10 (1:1000)

The same blot used for the anti-myc was used for the anti-whirlin mAb G-8 (1:1000)

Anti-GAPDH mAb (1:1000)

Supplementary Figure 4. Full scans of Western blots shown in Figures 6g,h

Blots for Fig. 6i

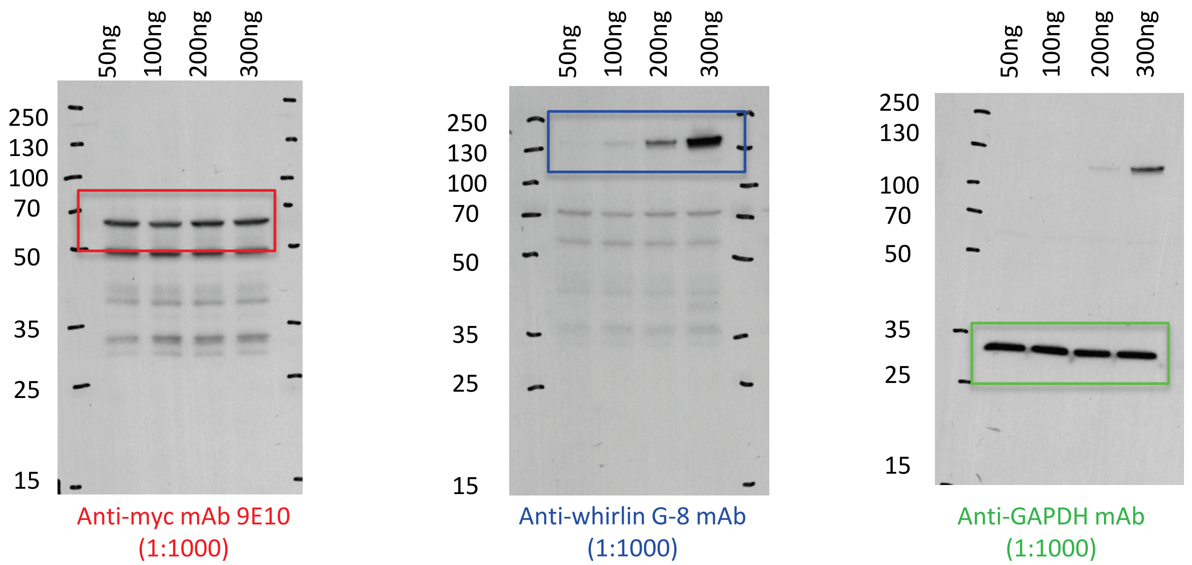

Blots for Fig. 8f

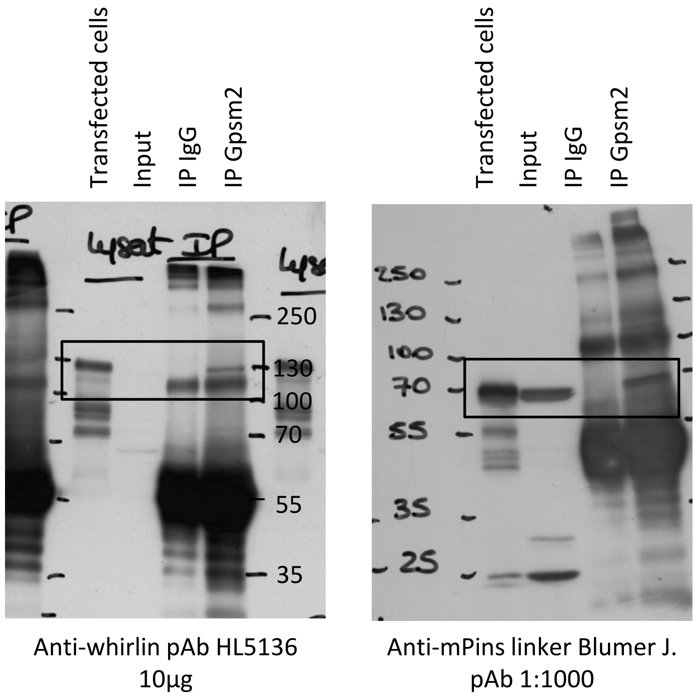

Supplementary Figure 5. Full scans of Western blots shown in Figures 6i and 8f.

Blot for Fig. 8h

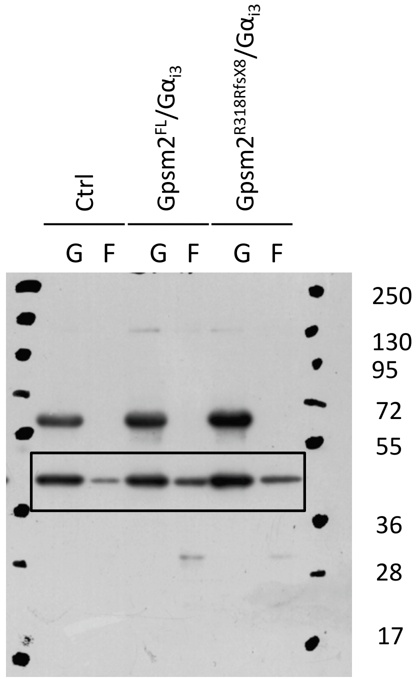

Anti-Actin pAb (1:500)

Blots for Fig. 8i

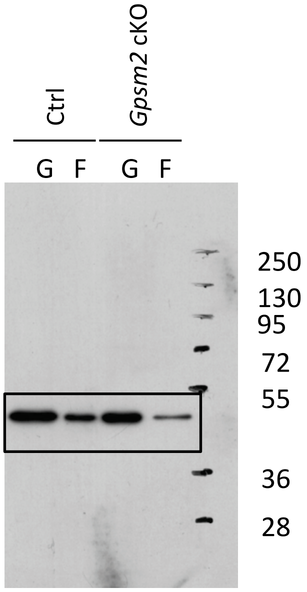

Anti-Actin pAb (1:500)

Blots for Supplementary Fig. 2a

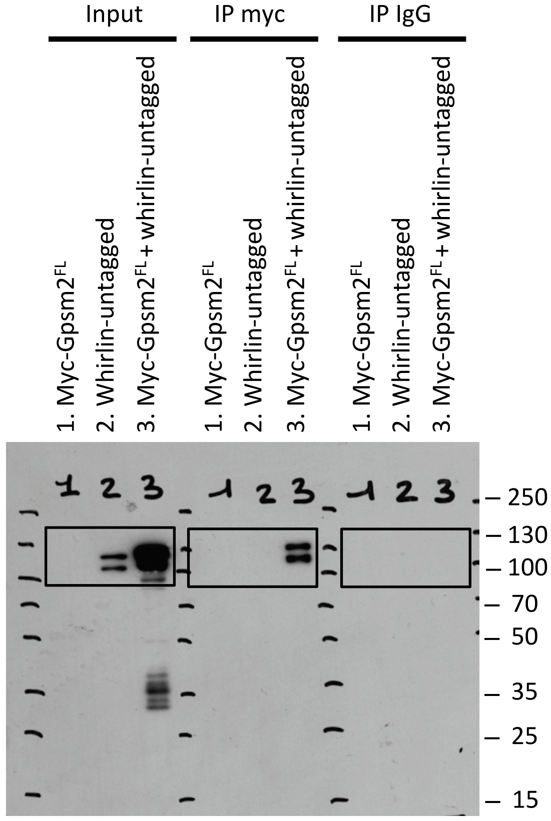

Anti-whirlin mAb G-8 (1:1000)
IP with anti-myc pAb Millipore / IgG Rabbit serum

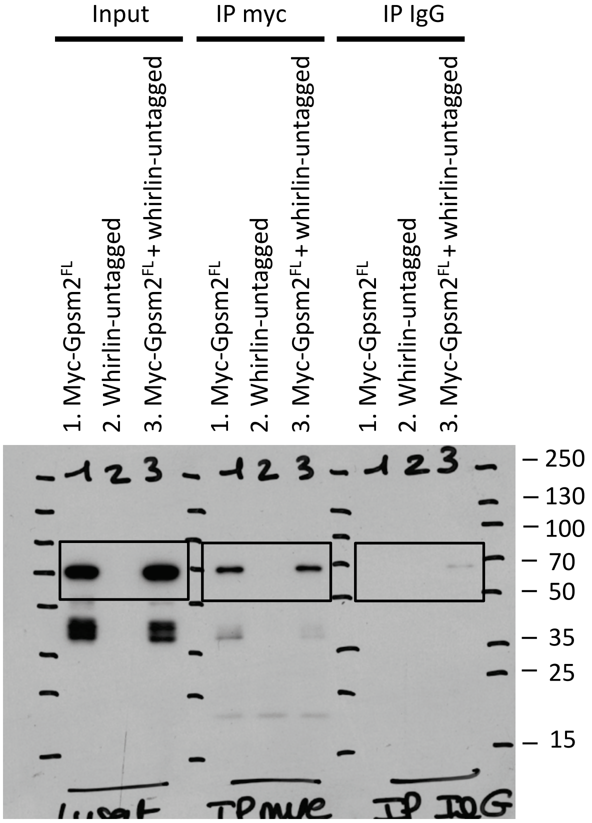

Anti-myc mAb 9E10 (1/1000)
IP with anti-myc pAb Millipore / IgG Rabbit serum

Supplementary Figure 6. Full scans of Western blots shown in Figures 8h, I and Supplementary Figures 2a

Blots for Supplementary Fig. 2b

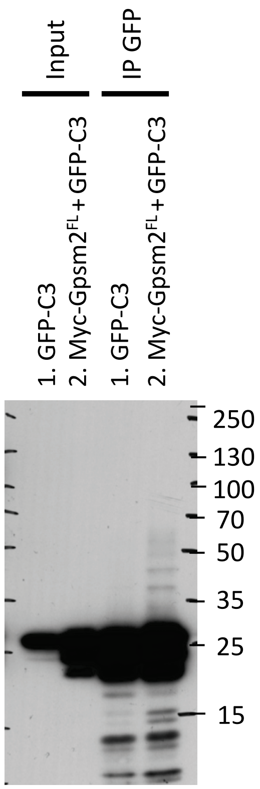

Anti-GFP mAb JL-8 (1:1000)
IP with IgG Rabbit serum

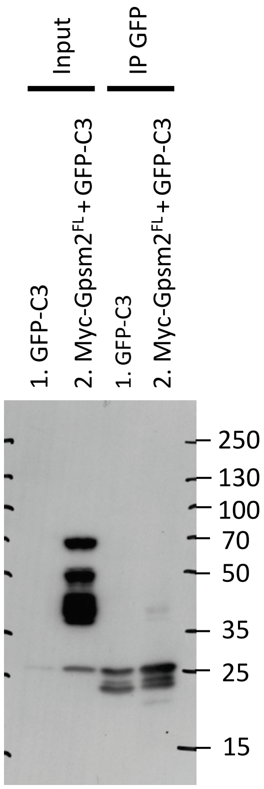

Anti-myc mAb 9E10 (1:1000)
IP with IgG Rabbit serum

Blots for Supplementary Fig. 2c

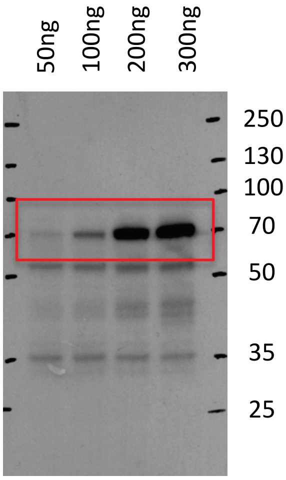

Anti-myc mAb 9E10
(1:1000)

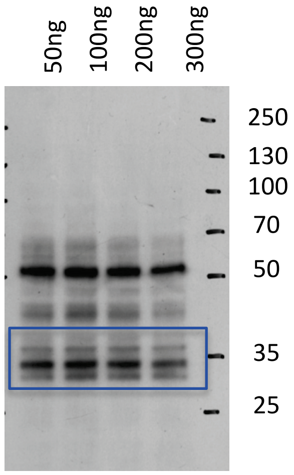

Anti DsRed mAb (1:500)

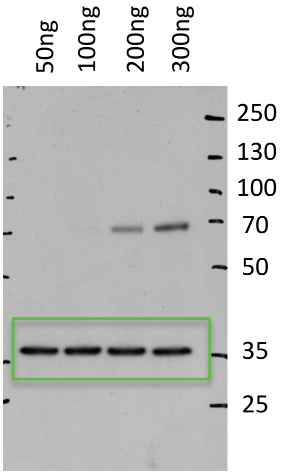

Anti-GAPDH mAb
(1:1000)

Supplementary Figure 7. Full scans of Western blots shown in Supplementary Figures 2b,c
